# An ortholog of *LEAFY* in *Jatropha curcas* regulates flowering time and floral organ development

**DOI:** 10.1038/srep37306

**Published:** 2016-11-21

**Authors:** Mingyong Tang, Yan-Bin Tao, Qiantang Fu, Yaling Song, Longjian Niu, Zeng-Fu Xu

**Affiliations:** 1 Key Laboratory of Tropical Plant Resources and Sustainable Use, Xishuangbanna Tropical Botanical Garden, Chinese Academy of Sciences, Menglun, Mengla, Yunnan 666303, China; 2 University of Chinese Academy of Sciences, Beijing 100049, China

## Abstract

*Jatropha curcas* seeds are an excellent biofuel feedstock, but seed yields of *Jatropha* are limited by its poor flowering and fruiting ability. Thus, identifying genes controlling flowering is critical for genetic improvement of seed yield. We isolated the *JcLFY*, a *Jatropha* ortholog of *Arabidopsis thaliana LEAFY* (*LFY*), and identified *JcLFY* function by overexpressing it in *Arabidopsis* and *Jatropha*. *JcLFY* is expressed in *Jatropha* inflorescence buds, flower buds, and carpels, with highest expression in the early developmental stage of flower buds. *JcLFY* overexpression induced early flowering, solitary flowers, and terminal flowers in *Arabidopsis*, and also rescued the delayed flowering phenotype of *lfy-15*, a *LFY* loss-of-function *Arabidopsis* mutant. Microarray and qPCR analysis revealed several flower identity and flower organ development genes were upregulated in *JcLFY*-overexpressing *Arabidopsis. JcLFY* overexpression in *Jatropha* also induced early flowering. Significant changes in inflorescence structure, floral organs, and fruit shape occurred in *JcLFY* co-suppressed plants in which expression of several flower identity and floral organ development genes were changed. This suggests *JcLFY* is involved in regulating flower identity, floral organ patterns, and fruit shape, although *JcLFY* function in *Jatropha* floral meristem determination is not as strong as that of *Arabidopsis*.

*Jatropha curcas* (hereafter *Jatropha*; Euphorbiaceae) is a perennial deciduous shrub that is widespread throughout the tropics and subtropics of central America^1^. *Jatropha* is monoecious, with male and female flowers borne on the same inflorescences^2^. The potential for *Jatropha* as a biofuel crop in tropical and subtropical countries is widely recognized^3^,^4^. *Jatropha* has been cultivated for its unique biodiesel potential because of its high oil content, high biomass productivity, adaptability to marginal land across various agro-climatic conditions, and non-competitiveness with food production^2^,^3^,^4^. This high agro-industrial potential for biofuel production, including bio-jet fuel production, has motivated cultivation of *Jatropha*^5^,^6^. The economic value of *Jatropha* is derived from its seed oil; *Jatropha* seeds contain up to 40% oil^7^. Accordingly, *Jatropha* cultivation may alleviate future energy crises and reduce environment pollution associated with petroleum production^8^. However, *Jatropha* exhibits an overabundance of vegetative shoots and leaves and a long juvenile stage^5^,^9^. In addition, unreliable and poor flowering is an important factor that contributes to low seed productivity in *Jatropha*. Breeders chiefly aim to shorten the juvenile phase; increase seed yield; reduce plant height; increase the ratio of female to male flowers; and improve oil content and oil fuel properties^5^,^7^. Thus, identifying flowering genes is one of the key steps to improve seed yield and enhance industrial use of *Jatropha*.

Flowering is the developmental turning point from the vegetative to reproductive phase and critical to crop yields. The precise timing of flowering is crucial to reproductive fitness, as plants must ensure the energy and resources accumulated during the vegetative phase are optimally allocated to offspring^10^. Both environmental and endogenous cues determine the onset of reproductive development^11^. Identification of flowering time genes is a key step for breeding plant varieties adapted to different agricultural conditions and seasons^10^. These signals modulate the level and activity of flowering-time regulators that initiate the reproductive phase and induce expression of the meristem identity genes^12^. Flowering has been most extensively studied in *Arabidopsis thaliana*, in which flowering is regulated by at least five parallel pathways: photoperiod, vernalization, gibberellin (GA)-dependent, autonomous, and age-related pathways^11^. *LEAFY* (*LFY*), *FLOWERING LOCUS T* (*FT*), and *SUPPRESSOR OF OVEREXPRESSION OF CONSTANS1* (*SOC1*)/*AGAMOUS-LIKE 20 (AGL20)* are genes that integrate signals from multiple genetic pathways^13^.

The *LFY* gene was identified by Weigel *et al*.^14^ in *Arabidopsis*, and its orthologous gene, *FLORICAULA* (*FLO*), was identified by Coen *et al*.^15^ in *Antirrhinum majus*. A single *LFY* ortholog is found in most land plant species, even in monocots whose evolutionary histories include several polyploidization events^16^. LFY is a plant-specific transcription factor that binds to the regulatory region of its target genes at a helix-turn-helix motif within a unique protein fold^17^. In seeds plants, *LFY* is the central flower meristem identity gene; it is mainly expressed in inflorescence and flower primordia^14^,^15^,^18^. *LFY* expression level is an important determinant of flower initiation^19^. LFY acts as a master regulator, orchestrating the floral gene network; it activates downstream genes that determine the unique identities of floral meristem (FM) tissue and floral organ primordia. LFY directly regulates flower organ identity genes including *CAULIFLOWER* (*CAL*), *APETALA1* (*AP1*), *APETALA3* (*AP3*), *AGAMOUS* (*AG*), *SEPALLATA* (*SEP*), and *TERMINAL FLOWER1* (*TFL1*)^20^,^21^. Accordingly, LFY controls multiple aspects of floral morphogenesis, including phyllotaxis, organ number, organ identity, and determinacy^22^. LFY executes its meristem identity role by activating *AP1* expression, as is supported by the recovery of the *lfy* mutant phenotype by overexpression of *AP1*^22^. LFY is also involved in inflorescence and flower development. In maize (*Zea mays*), LFY homologs ZFL1 and ZFL2 are required for proper expression of B and C genes in flowers^23^. In contrast, the LFY homolog in rice, RFL, controls inflorescence structure; RFL is unexpressed in FMs, and rice flowers appear fertile even when RFL is silenced^24^. LFY homologs have an ancestral role in regulating cell division and arrangement in gymnosperms, ferns, and mosses—taxa that lack flowers^17^. In pea and *Medicago truncatula*, LFY orthologs are involved in leaf development and are required to form leaflets^25^,^26^.

*LFY* overexpression in *Arabidopsis* induces early flowering and converts all buds into flower buds while making all inflorescences occur as solitary and terminal flowers^19^,^27^. Additionally, *LFY* overexpression accelerates flowering in a variety of other plants, including tobacco^28^, rice^24^, S*inningia speciosa*^29^, *Brassica juncea*^30^, and *Citrus*^31^. In *Arabidopsis lfy* mutants, FM development is dramatically delayed with late flowering^32^, flower buds are surrounded by extra bracts, and the early flowers are completely transformed into inflorescence shoots. Additionally, the first four floral organs develop into sepal-like organs and develop fewer petals and stamens. Flowers produced by plants with intermediate alleles are often male sterile, but female fertile^14^. In *Antirrhinum*, mutations in *flo* transform flowers into inflorescence shoots completely, and these inflorescences exhibited meristems that proliferated without producing flowers^15^.

Several studies have reported that LFY interacts with hormone pathways. For example, LFY controls auxin biosynthesis through repressing the auxin synthesis gene *YUC4*; in turn, auxin increases *LFY* transcription, and LFY promotes auxin signaling, leading to further increases in *LFY* activity^33^. In *Arabidopsis*, GA regulates *LFY* transcription to promote flower initiation^34^,^35^,^36^. LFY represses the cytokinin signaling negative regulator *ARR7* through a direct interaction with its promoter^20^.

*LFY* orthologs have been cloned and characterized in several woody species such as *Eucalyptus*^37^, Monterey pine^38^, Kiwifruit^39^, and papaya^40^. However, few is known about the specific role of *LFY* in tree reproductive development. In woody species, juvenile phases can last years to decades such that trees delay flowering and fruit production for a very long time. The existence of a lengthy juvenile phase in woody species is a limiting factor for their genetic improvement, and this prevents the full domestication of many economically important woody species. Currently, only *Jatropha FLOWERING LOCUS T* (*JcFT*)^41^,^42^, and *JcAP1*^43^ have been functionally analyzed in *Jatropha*. However, cloning and molecular characterization of *JcLFY* has not yet been reported. In this study, we cloned *JcLFY* from *Jatropha* and analyzed the function of *JcLFY* by transforming both wild-type (WT) and *lfy*-*15* mutant *Arabidopsis*. We further analyzed *JcLFY* function in flowering induction and floral organ specification using *JcLFY* overexpression and co-suppression in transgenic *Jatropha* plants.

## Results

### Cloning and bioinformatic analysis of *JcLFY* in *Jatropha*

Full length *JcLFY* cDNA sequence was obtained from *Jatropha* flower bud tissue by RT-PCR and RACE. *JcLFY* gDNA and cDNA were amplified by long-distance PCR. The full 2302-bp length of *JcLFY* gDNA contains two introns and three exons, and the full 1350-bp length of *JcLFY* cDNA contains a 1155-bp open reading frame, encoding 384 amino acids (Supplementary Fig. S1A). Alignment of the JcLFY sequence with *Ricinus communis* LFY (RcLFY), *Populus trichocarpa* LFY (PtLFY), *Arabidopsis thaliana* LFY (AtLFY), *Oryza sativa* FLO (OsFLO), and *Zea mays* FLO (ZmFLO) showed amino acid sequence identities of 88%, 84%, 72%, 69%, and 66%, respectively. LFY proteins show higher similarity in the C-terminal region (Supplementary Fig. S1B).

A total of 41 LFY amino acid sequences from different species were used to construct a phylogenetic tree showing the relationship of *LFY* homologs (Supplementary Fig. S2). These genes can be classified into five classes, which generally differentiate lower plants from higher plants (Supplementary Fig. S2). Specifically, two genes from mosses form class I; ten genes from ferns form class II; five genes from gymnosperms form class III; fifteen genes from eudicots form class IV; and nine genes from monocots form class V (Supplementary Fig. S2). JcLFY clustered together with sequences from other eudicots plants and belongs to class IV, and JcLFY has the highest identity to RcLFY from *Ricinus communis* (Supplementary Fig. S2). RcLFY and JcLFY clustered in the same clade, consistent with the close evolutionary relationship between *Jatropha* and *Ricinus communis*.

### Expression pattern of *JcLFY* in *Jatropha*

**Figure 1:**
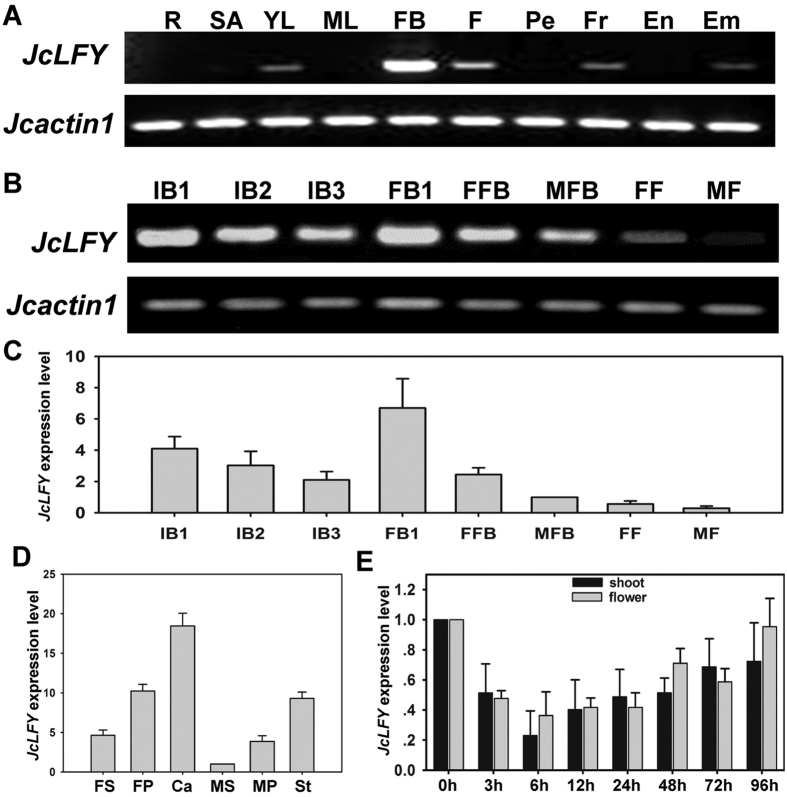
Analysis of *JcLFY* expression in different *Jatropha* tissues. (**A**,**B**) Semi-quantitative RT-PCR analysis of *JcLFY* expression in different tissues; (**C**) Real-time qPCR performed on inflorescence buds and flower buds to validate semi-quantitative RT-PCR results; (**D**) Real-time qPCR analysis of *JcLFY* expression in floral organs; (**E**) Real-time qPCR analysis of *JcLFY* expression in shoot and flower bud tissues after 1 mM GA application. R, root; SA, shoot apex; YL, young leaf; ML, mature leaf; FB, flower bud; F, flower; Pe, pedicel; Fr, fruit; En, endosperm; Em, embryo; FS, female sepal; FP, female petal; Ca, carpel; MS, male sepal; MP, male petal; St, stamen; IB1, inflorescence bud stage 1 (0–5 days, inflorescence buds are visible); IB2, inflorescence bud stage 2 (1 week after IB1); IB3, inflorescence bud stage 3 (1 week after IB2); FB1, flower bud stage 1 (1 week after IB3); and FB2, flower bud stage 2 (1 week after FB1). In this stage, male flower bud (MFB) and female flower bud (FFB) are identifiable: MF, male flower (1 week after MFB) and FF, female flower (1 week after FFB). Fruits were harvested 15 days after fertilization. Endosperm and embryo tissues were harvested from mature seeds. The levels of detected amplicons were normalized using the amplified products of *JcActin1*. The mRNA levels in FFB (**C**), MS (**D**), and before GA application to FB (**E**) tissues were standardized with a value of 1. The total PCR cycles of *JcActin1* in (**A**) and (**B**) are 26 and 25, respectively, while the PCR cycles of *JcLFY* in (**A**) and (**B**) are both 30.

RT-PCR was used to investigate the expression of *JcLFY* across different organs and tissues. We first tested the expression of *JcLFY* in various tissues, revealing that this gene is highly expressed in flower buds and the transcript occurs in young leaves, flowers, fruits, and embryos. However, transcripts were not detected in roots, shoot apices, mature leaves, pedicels, and endosperms (Fig. 1A). To better analyze *JcLFY* transcripts, samples were collected from flowers at different developmental stages (Fig. 1B). *JcLFY* was highly expressed in inflorescence buds (IB1, IB2, and IB3), flower buds (FB), male flower buds (MFB), and female flower buds (FFB), but exhibited lower expression levels in bloomed male and female flowers (Fig. 1B). Overall, *JcLFY* was highly expressed during the early-stage inflorescence buds (e.g., IB1) and early-stage flower buds (e.g., FB1). During inflorescence and floral organ development, *JcLFY* expression levels decreased. Real-time qPCR agreed with the semi RT-PCR results of *JcLFY* expressions across flower development stages (Fig. 1C). We further analyzed *JcLFY* expression in different floral whorls, finding *JcLFY* expression higher in stamens and carpels than in sepals and petals (Fig. 1D). To determine whether *JcLFY* was induced by GA, we assessed *JcLFY* expression levels in shoot apex and flower bud tissues after application of 1 mM GA. Unexpectedly, *JcLFY* expression exhibited a slight decrease after GA application (Fig. 1E). Hence, *JcLFY* was not induced by GA in *Jatropha*, in contrast with *Arabidopsis*^34^,^35^ and *Chrysanthemum*^44^.

### *JcLFY* overexpression in *Arabidopsis* induced early flowering, solitary flowers, and terminal flowers

To determine whether *JcLFY* is involved in the regulation of flowering time, *JcLFY* cDNA driven by the CaMV 35S promoter was transformed into WT *Arabidopsis* (untransformed WT plants were controls). Transgenic plants were confirmed via qRT-PCR analysis of *JcLFY* expression. Forty independent T0 transgenic lines were generated with the 35S:*JcLFY* construct. In a majority of transgenic lines, bolting occurred notably earlier than in WT plants under both LD and SD conditions.

**Figure 2:**
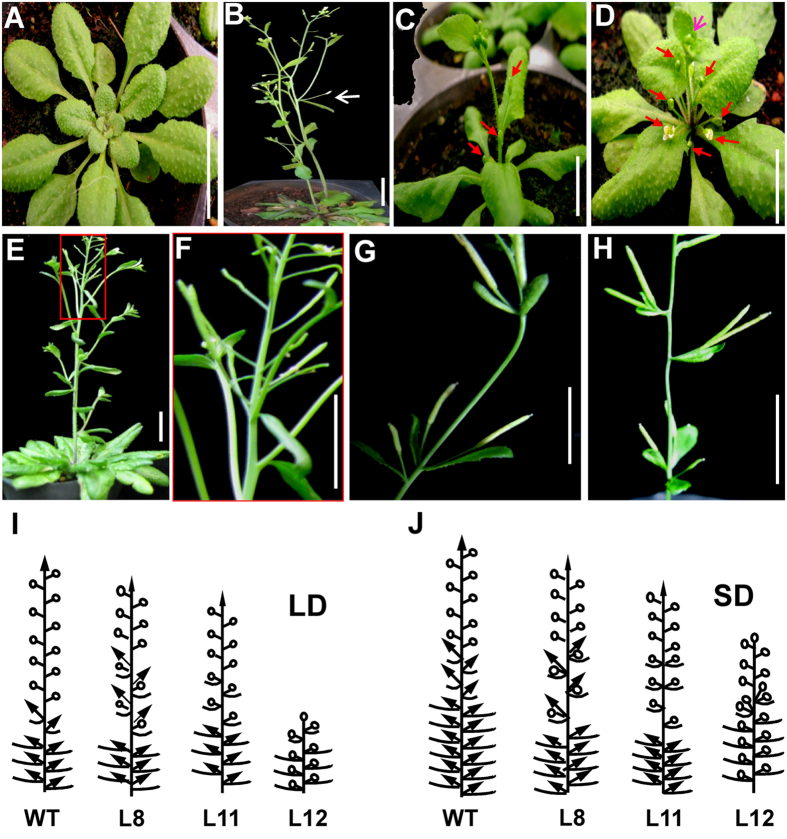
Phenotypes of transgenic *Arabidopsis* overexpressing *JcLFY*. (**A**) Wild-type (WT) *Arabidopsis* under LD conditions (30 d). (**B**) Transgenic *Arabidopsis* L8 grown under LD conditions (40 d); the white arrow indicates a branch and a solitary flower formed in a cauline leaf axil. (**C,D**) Transgenic *Arabidopsis* L11 and L12 grown under LD conditions (20 d); the red arrows indicate the solitary flowers, while the pink arrow indicates the terminal flower. (**E**) WT *Arabidopsis* grown under SD conditions (120 d). (**F**) Detailed image of contents of the red box in (**E**). (**G**) Transgenic plant L11 grown under SD conditions, exhibiting opposite leaves and solitary flowers formed in leaf axils; fruit development was normal. (**H**) Transgenic plant L12 grown under SD conditions; two flowers formed in a leaf axil and fruit development was normal. All *Arabidopsis* plants were derived from the Columbia ecotype. (**I**,**J**) Schematic comparison of WT and 35S:*JcLFY* transgenic *Arabidopsis* plants grown under LD and SD conditions, respectively. Arrowheads indicate shoot meristems, while circles indicate flowers. In WT plants, the primary shoot can be subdivided into a basal rosette, which contains leaves separated by short internodes, and an apical shoot with elongated internodes; the apical shoot, often referred to as an inflorescence, bears a few bracts (small stem leaves) with associated secondary shoots, as well as a potentially indeterminate number of flowers. Scale bar = 1 cm.

The phenotypes of three independent homozygous lines from the T2 generation were examined. Relative to WT plants under LD conditions, L8, L11, and L12 bolted 9–15 days earlier, produced 2–6 fewer rosette leaves, produced 2–4 fewer branches, and were 3–17.5 cm shorter (Fig. 2B–D and Table 1). Under SD conditions, L8, L11, and L12 flowered approximately 1–2 months earlier, produced 16–36 fewer rosette leaves, and were 6.1–32.7 cm shorter (Table 2). Thus, *JcLFY* overexpression in *Arabidopsis* significantly reduced the vegetative growth period.

**Figure 3:**
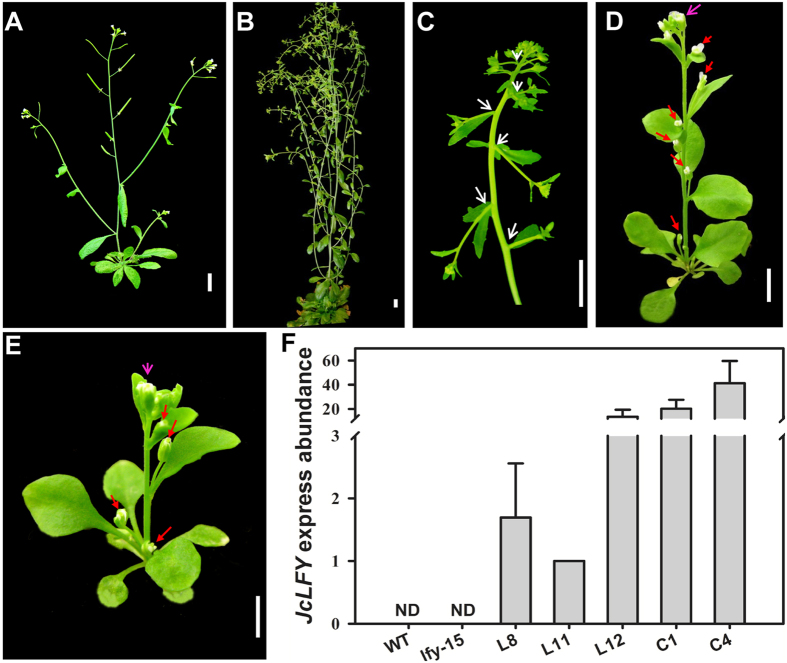
35S:*JcLFY* recovered *lfy-15* mutant *Arabidopsis* phenotypes. (**A**) WT *Arabidopsis* (45 d). (**B**) *lfy-15* mutant (70 d). (**C**) Inflorescence of a *lfy-15* mutant with flowers transformed into inflorescences/branches (white arrows). (**D**) *lfy-15* mutant harboring 35S:*JcLFY* transgenic plant C1 (25 d). (**E**) *lfy-15* mutant harboring 35S:*JcLFY* transgenic plant C4 (20 d). All *Arabidopsis* were derived from the Columbia ecotype. The red arrows in (**D**) and (**E**) indicate solitary flowers; the pink arrows in (**D**) and (**E**) indicate terminal flowers (scale bar = 1 cm). (**F**) Data generated by qRT-PCR were used to quantitatively assess the expression of *JcLFY* transgenes. Expression profiles were normalized against *AtActin2*. Expression analysis was conducted using quantitative RT-PCR with RNA extracted from seedlings of T2 transgenic L8, L11, L12, and *lfy-15* mutants harboring *JcLFY* (C1 and C4). WT and *lfy-15* plants grown under the same conditions were used as controls; *JcLFY* expression was not detected in both WT and *lfy-15* mutant. Error bars represent the standard deviation (SD) across three independent replicates.

In transgenic plants, the primary shoots were converted into terminal flowers (Fig. 2D). The secondary shoots produced in cauline and rosette leaf axils were converted into solitary flowers. In extreme phenotypes of transgenic plants, all branches and inflorescences were replaced by solitary flowers (Fig. 2C,D). Furthermore, transgenic plants under SD conditions exhibited opposite leaves, solitary flowers in leaf axils (Fig. 2G), and two flowers within a single leaf axil (Fig. 2H). Transgenic plants L12 exhibited the highest *JcLFY* expression levels (Fig. 3F) and the most severe phenotypes (Fig. 2D).

To explore genes involved in the *JcLFY* mediated pathway, 2-week-old soil-grown transgenic 35S:*JcLFY* L8, L11, and L12 plants as well as WT *Arabidopsis* were grown under LD conditions for microarray analysis. In total, 1,854 genes exhibited more than 2-fold changes, 122 genes exhibited more than 10-fold changes, and 22 genes exhibited more than 50-fold changes (Supplementary Table S3, and Fig. S4A). We classified 2-fold or higher expression changes in these three lines into six groups. Overall, 48 flower-related genes were detected by microarray at this threshold (Supplementary Fig. S4); 71 phytohormone-related genes changed expression, including genes related to auxin, cytokinin, gibberellin, abscisic acid, brassinosteroids, and jasmonate; 58 stress-related genes were changed; 480 genes encoding metabolic enzymes were changed; and 474 genes of unknown function were changed (Supplementary Table S3 and Fig. S4B).

Microarray data and qRT-PCR results indicated the promotion of flowering and terminal flowers in 35S:*JcLFY* transgenic *Arabidopsis* was associated with a significant upregulation of the FM identity genes *AP1, SOC1*, and *CAL*, and the floral organ identity genes *AG, AP3*, *SEP1*, *SEP2*, and *SEP3* (Supplementary Fig. S3). The expression levels of these genes were highest in L12. *TFL1* expression was downregulated in L12 plants (Supplementary Fig. S3), which likely induced solitary terminal flowers. Thus, the early-flowering and terminal flower phenotypes induced by ectopic *JcLFY* expression in transgenic *Arabidopsis* were similar to those induced by *AtLFY* overexpression^27^. Under both LD and SD conditions, *JcLFY* overexpression in *Arabidopsis* induced early flowering.

Chromatin immuno-precipitation experiment^45^ showed *Arabidopsis LFY* has 15 direct target genes, and each target gene was upregulated more than 2-fold. However, most of these target genes were upregulated in the present study, though the fold changes were much lower (e.g., AT5G60630, AT5G49770, At5G03790, AT5G03230, and At2g44450 in Supplementary Table S2). Some of these target genes exhibited no significant change (e.g., AT4G22780 and AT5G46660), while others were downregulated (e.g., AT3G52470 and AT3G19390) Supplementary Table S2.

### *JcLFY* overexpression in *lfy-15* mutant *Arabidopsis* induced early flowering and complemented mutant phenotypes

To further determine whether *JcLFY* is an *AtLFY* ortholog, the 35S:*JcLFY* construct was transformed into *Arabidopsis lfy-15* mutant plants. Ten independent T0 transgenic lines were generated and confirmed through qRT-PCR of *JcLFY* expression. WT plants and *lfy-15* mutants under the same growth conditions were controls. Most transgenic lines bolted earlier than the WT and *lfy-15* mutant plants under LD conditions.

To examine phenotypes, we selected two independent homozygous lines in the T2 generation that exhibited high *JcLFY* expression (Fig. 3D,E). Under LD conditions, lines C1 and C4 bolted 8–13 days earlier and produced 2–6 fewer rosette leaves than WT plants, and bolted 18–23 days earlier and produced 9–13 fewer rosette leaves than *lfy-15* mutants (Fig. 3A–E; Table 3). In transgenic plants, solitary flowers appeared in the axils of rosette and cauline leaves, and terminal flowers appeared on primary shoots (Fig. 3D,E). The *lfy-15* mutant flowers were converted to inflorescences, and floral organs were abnormal and less fertile (Fig. 3C)^14^. The transgenic mutant C1 and C4 lines rescued mutant late bolting, and the conversion of flowers to inflorescences was repressed (Fig. 3D,E).

Both C1 and C4 exhibited high *JcLFY* expression (Fig. 3F). Promotion of flowering in the 35S:*JcLFY* transgenic *Arabidopsis* mutant was associated with a significant up-regulation of FM identity genes *AP1*, *SOC1*, and *SEPs* (Supplementary Fig. S3). The branch reduction may have occurred through the repression of *TFL1* by *JcLFY* (Supplementary Fig. S3I).

These results demonstrate that the constitutive expression of *JcLFY* complements increased branches (i.e., inflorescences) in later flowering stages and abnormal flowers in *lfy-15* mutant; thus, *JcLFY* functions as a *LFY* homolog.

### *JcLFY* overexpression in *Jatropha* induced early flowering

**Figure 4:**
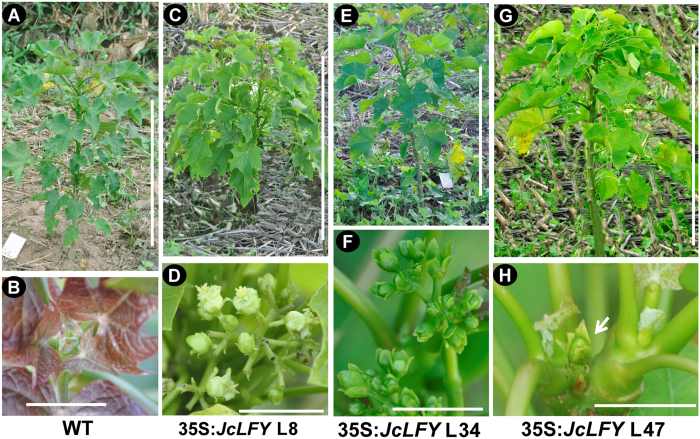
35S:*JcLFY*-overexpressing transgenic *Jatropha* exhibited slightly early flowering (**A**) Wild-type (WT) *Jatropha* grown in the field for 2 months and still in the vegetative stage, bar = 50 cm. (**B**) Shoot apex of WT *Jatropha* in the field, no flower produced. bar = 1 cm. (**C**,**E**,**G**) 35S:*JcLFY* transgenic *Jatropha* (L8, L34, and L47, respectively) grown in the field for 2 months and at the anthesis stage, bar = 50 cm. (**D**,**F**,**H**) Inflorescence of transgenic *Jatropha* in the field with a single terminal flower on L47 (white arrow indicated in H). bar = 1 cm.

*Jatropha* expression profiles and *Arabidopsis* transgenic analyses suggested *JcLFY* is a floral identity gene in *Jatropha*. To test this hypothesis, we generated transgenic *Jatropha* with the 35S:*JcLFY* construct. Non-transgenic plants were used as a control. Fifty independent transgenic lines were confirmed via PCR. Unexpectedly, most transgenic *Jatropha* plants lacked an early-flowering phenotype, while some transgenic plants (approximately 10) showed slightly early flowering. When regenerated plantlets (Fig. 4) were grown in the field for 2 months, flower buds emerged from transgenic plants (Fig. 4C–H), while control plants didn’t produce flower buds (Fig. 4A,B). We chose early-flowering plants from L8, L34, and L47 for further analysis. qRT-PCR indicated *JcLFY* expression was higher in these plants, while *JcLFY* expression was much higher in the L47 sample (Supplementary Fig. S5A). We further analyzed several floral identity genes and flower organ identity genes, finding that the transcript levels of *JcAP1, JcAP3*, *JcAG*, *JcSEP1*, and *JcSEP3* were obviously increased in transgenic flower buds; however, *JcAP2* and *JcTFL1c* expression was reduced. The inflorescence structure and floral organ patterns were normal. Furthermore, we grew 15 transgenic plants in a greenhouse in Kunming located in a subtropical area of southwestern China (Supplementary Fig. S6). We found 2 transgenic plants produced flowers in the second spring, and 5 transgenic plants produced flowers in the third spring (Supplementary Fig. S6). However, the WT plants didn’t produce flowers up to five years after plantation in the greenhouse (Supplementary Fig. S6). The results obtained from the transgenic *Jatropha* plants indicate that *JcLFY* is involved in FM determination in *Jatropha*.

### *JcLFY* co-suppression changed inflorescence structure and flower organ pattern

**Figure 5:**
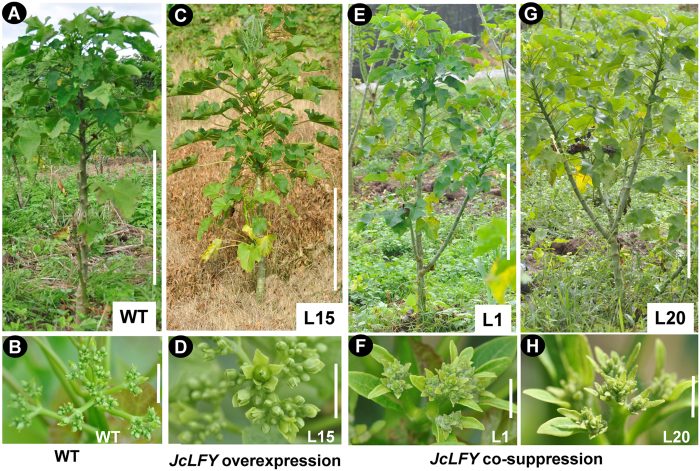
Co-suppression of *JcLFY* in *Jatropha* led to severe deficiencies in inflorescences and flowers. (**A**) Wild type (WT) grown in the field for 4 months (at the anthesis stage), bar = 50 cm. (**B**) WT inflorescence in (**A**), bar = 1 cm. (**C**) 35S:*JcLFY* transgenic *Jatropha* L15 grown in the field for 4 months (at the anthesis stage); the plants exhibited normal flowering times and floral organs, bar = 50 cm. (**D**) Transgenic *Jatropha* inflorescence from L15 in (**C**), bar = 1 cm. (**E**,**G**) 35S:*JcLFY* co-suppressed *Jatropha* (L1 and L20) grown in the field for 4 months; plants exhibited normal flowering times, bar = 50 cm. (**F**,**H**) Inflorescences of L1 in (**E**) and L20 in (**G**), these plants exhibited abnormal inflorescences. bar = 1 cm.

Among transgenic *Jatropha* generated with the 35S:*JcLFY* construct, we found three *JcLFY* co-suppressed plants. qRT-PCR results showed that *JcLFY* expression levels were more than 10 folds lower than WT in flower buds; L1 exhibited the lowest *JcLFY* expression levels (Supplementary Fig. S5A). Such co-suppression was first reported by Napoli *et al*.^46^, and it has been widely reported in many transgenic plants and animals^47^. These co-suppressed transgenic *Jatropha* plants exhibited no late-flowering phenotypes. When regenerated plantlets were grown in the field for 4 months, flower buds emerged in both co-suppressed transgenic and control plants (Fig. 5A,C,E,G).

Unlike many other species, *Jatropha* flowers are not subtended by small leaves called bracts (Fig. 5B,D). *Jatropha* flowers are composed of three concentric rings of organs: five sepals in the first, outermost whorl; five petals in the second whorl; and ten stamens (male flower) or one carpel (female flower) in the third whorl.

Inflorescences of *JcLFY* co-suppressed plants exhibited many bracts surrounding florets (Fig. 5F,H). One primary inflorescence branch was analyzed for its secondary inflorescence structure revealing there were more secondary inflorescence branches in co-suppressed plants.

**Figure 6:**
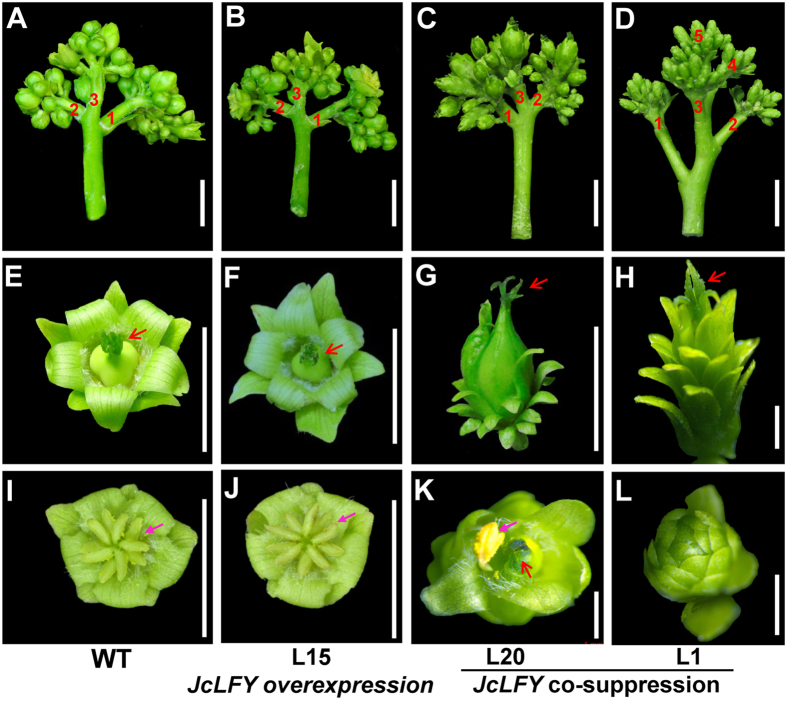
Phenotypes of co-suppressed *JcLFY Jatropha* flowers. (**A**) A primary inflorescence branch from a wild-type (WT) plant; 3 secondary inflorescence branches (noted by numerals) were produced (bar = 1 cm, unless otherwise noted). (**B**) A primary inflorescence branch of *JcLFY* overexpression plant (L15); 3 secondary inflorescence branches (noted by numerals) were produced. (**C**) A primary inflorescence branch of a *JcLFY* co-suppressed plant (L20); 3 secondary inflorescence branches (noted by numerals) were produced. (**D**) A primary inflorescence branch of *JcLFY* co-suppressed plant (L1); 5 secondary inflorescence branches (noted by numerals) were produced. (**E**) Female flower (central flower) of WT plant. (**F**) Female flower (central flower) of *JcLFY*-overexpression plants (L15). (**G**) Female flower (central flower) of *JcLFY* co-suppression plant (L20). (**H**) Central flower of *JcLFY* co-suppressed plant (L1, bar = 1 mm). (**I**) Male flower of WT plant. (**J**) Male flower (marginal flower) of *JcLFY* overexpression plants (L15). (**K**) Marginal flower of *JcLFY* co-suppressed plant (L20) with 1 stamen and 1 stigma exhibited in the same flower (bar = 1 mm). (**L**) Marginal flower of a *JcLFY* co-suppressed plant (L1); the flower could not bloom (bar = 1 mm). Red arrows indicate stigmas; pink arrows indicate stamens.

**Figure 7:**
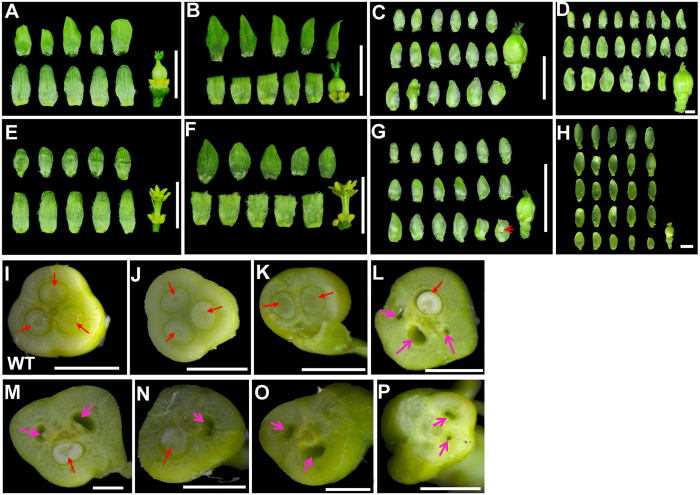
Anatomy of flowers from *JcLFY* overexpression and co-suppressed plants. (**A**) Wild-type (WT) female flower (bar = 1 cm, unless otherwise noted). (**B**) *JcLFY* overexpression female flower. (**C**) *JcLFY* co-suppression female flower (L20) with 15–20 sepaloid organs in each flower. (**D**) *JcLFY* co-suppression female flower (L1) with 20–25 sepaloid organs in each flower (bar = 1 mm). (**E**) WT male flower. (**F**) *JcLFY* overexpression male flower. (**G**) *JcLFY* co-suppressed marginal flower (bisexual flower; L20) with a stamen fused to a sepaloid organ (red arrow). There are 15–20 sepaloid organs in each flower. (**H**) *JcLFY* co-suppressed marginal flower (no ovule, female flower; L20). There are 22–28 sepaloid organs in each flower (bar = 1 mm). (**I**) WT carpel cross section with three ovules (bar = 1 mm). (**J**) *JcLFY* overexpression carpel cross section with three ovules (bar = 1 mm). (**K**–**P**) *JcLFY* co-suppressed carpel cross section with ovules and the ovule cavity partially or completely absent (bar = 1 mm). Red arrows indicate ovules and pink arrows indicate the ovule cavities without ovules.

Flowers of weakly *JcLFY* co-suppressed plants (i.e., L20) bloomed, but all floral organs were abnormal. In male and female flowers, sepals and petals were replaced by sepal-like structures (sepaloid organs). In female flowers, stigmas were abnormal (Fig. 6G); in male flowers, only 1–2 stamens were observed, but abnormal stigmas also occurred (Fig. 6K). The first few flowers were more abnormal than later flowers; most of the early arising flowers were aborted. Such female flowers (the central flowers, specifically) have 15–20 sepaloid organs and an abnormal carpel (Fig. 7C); marginal flowers (bisexual flowers; in WT, the flower in this position is specifically male) of L20 had 15–20 sepaloid organs and a stamen fused to sepaloid organs in each flower (Fig. 7C). A cross section of the carpels of this plant revealed abnormal ovule and ovule cavity numbers, with only 0–2 observable ovules (Fig. 7K–M). However, WT and *JcLFY*-overexpression plants exhibited three ovules and ovule cavities per flower (Fig. 7I,J). Since well-developed stamens are rare and ovule numbers were reduced, the fertility of weakly *JcLFY* co-suppressed plants was likely reduced.

Flowers of strongly *JcLFY* co-suppressed plants (i.e., L1) were all aborted, all flower organs were abnormal, and the flowers were smaller than those of the controls (Fig. 6D). Male and female flowers’ sepals and petals were replaced by sepaloid organs. Female flower stigmas were severely abnormal (Fig. 6H); in male flowers, stamens were unobserved (Fig. 6L). We dissected the flowers of such plants, finding the female flowers (i.e., central flowers) had 20–25 sepaloid organs and abnormal carpels (Fig. 7D); the marginal flower (in WT, this is a male flower) of L1 plants had 22–28 sepaloid organs and an abnormal carpel-like organ (Fig. 7H). Cross sections of the carpels of this plant revealed abnormal ovule and ovule cavity numbers, with only 0–1 ovules in central flowers (Fig. 7N,O) and no ovules in marginal flowers (Fig. 7P). Flowers of this plant were male and female sterile.

Comparison of these *JcLFY* co-suppressed plants demonstrated that stronger *JcLFY* co-suppression was associated with more sepaloid organs and fewer stamens and ovules. This indicates that *JcLFY* is important in regulating floral organ development.

### Altered expression of *JcLFY* affected fruit and seed development

**Figure 8:**
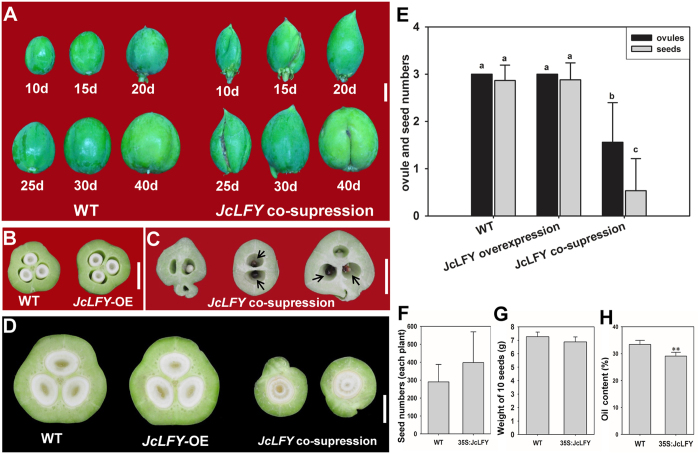
Fruit and seed development in *JcLFY* co-suppressed and *JcLFY* overexpressing transgenic plants. (**A**) Wild-type (WT, left) and *JcLFY* co-suppressed (L20, right) fruit at different development stages (10 d, 15 d, 20 d, 25 d, 30 d, and 40 d after fertilization); the fruits varied in size and shape; (**B**) WT (left) and *JcLFY* overexpression (right) fruit cross sections (15 d after fertilization). (**C**) Cross section of *JcLFY* co-suppressed fruits (10 d after fertilization). Most of the seeds were aborted (black arrows). (**D**) Cross section of WT, *JcLFY* overexpressing, and *JcLFY* co-suppressed fruits (40 d after fertilization). (**E**) Ovule and seed numbers in WT, *JcLFY* overexpression, and *JcLFY* co-suppression fruits. Each value is the mean of 30 fruits. Error bars represent standard deviations. Means with different letters are different according to Tukey’s tests (*p* < 0.05). Comparison of the seed number per plant (**F**), weight of 10 seeds (**G**) and seed oil content (**H**) between 35S:*JcLFY* T0 transgenic plants and WT. Fifteen plants from WT and T0 transgenic plants respectively were used for statistical analysis. The plants were 1-year old after transfer to the field. Scale bars = 1 cm. **Significantly different from the control at the 1% level.

To further analyze whether *JcLFY* can affect fruit development, we analyzed fruit phenotypes at various developmental stage. In *JcLFY* co-suppressed plants, we found fruits were longer than in WT plants. Additionally, fruits were narrower, and the shapes were severely abnormal, which was maintained to maturity (Fig. 8A). Cross sections of 10-day-old fruits revealed the WT and *JcLFY*-overexpression plants had fruits with 3 seeds each (Fig. 8B); however, the *JcLFY* co-suppressed plant fruits had only 0–1 seed each, and most of the seeds aborted at an early stage (Fig. 8C). Normally, seedless-fruits are aborted at an early stage, and most developable fruits have only one seed in each fruit (Fig. 8D,E). Because of ovule abortion in co-suppressed plants, number of seeds per fruit was fewer than number of ovules per female flower (Fig. 8E).

To assess the potential of *JcLFY*-overexpression plants in the genetic improvement of *Jatropha*, we analyzed the seed number per plant, seed weight and seed oil content in T0 transgenic plants of 35S:*JcLFY* (Fig. 8F–H). There was no statistical difference in seed number per plant between transgenic plants and wild-types, although the average seed number per plant of 35S:*JcLFY* was slightly more than that of wild-type (Fig. 8F). There was also no significant change in seed weight (Fig. 8G), but oil content in seeds of 35S:*JcLFY* transgenic plants was significantly decreased (Fig. 8H). Because extensive variation in seed number per plant was observed among T0 transgenic plants of 35S:*JcLFY* (Fig. 8F), homozygous plants from the transgenic lines by self-pollination and subsequent vegetative propagation need to be used for further assessment of the overall effect of *JcLFY*-overexpression on oil yield of transgenic plants.

## Discussion

### *JcLFY* is an ortholog of *Arabidopsis LFY*

JcLFY shares high amino acid sequence similarity with other LFY proteins and most closely resembles *Ricinus communis* LFY. JcLFY contains conserved domains such as a proline-rich region and a leucine zipper motif in the N-terminal domain (Supplementary Fig. S1B), suggesting that JcLFY might have a similar function as LFY. We detected *JcLFY* transcripts in several tissues, finding the highest accumulation in flower buds (Fig. 1A). Moreover, *JcLFY* expression was highest in early stage inflorescence buds (IB1) and early stage flower buds (FB1) (Fig. 1B,C), implying a possible role of *JcLFY* in regulating *Jatropha* flowering.

The present study has shown that over-expressing 35S:*JcLFY* in *Arabidopsis* can inhibit vegetative growth, hence reducing branch number and heights of transgenic plants while promoting early flowering, by approximately 15 days and 2 months relative to WT plants under LD and SD conditions, respectively (Tables 1 and 2). This is quite similar to phenotypes under constitutive *LFY* ortholog expression in *Arabidopsis*^27^, *Gloxinia*^29^, *Brassica juncea*^30^, and *Nicotiana tabacum*^28^. Constitutive *LFY* expression driven by the CaMV35S promoter attenuates development of both vegetative and inflorescence phases, inducing the production of terminal flowers on *Arabidopsis* primary shoots^27^.

This study has shown that *JcLFY* overexpression in *Arabidopsis* produced terminal and solitary flowers (Fig. 2). These findings are similar to the phenotypic changes caused by constitutive *LFY* expression in *Arabidopsis*^27^,^48^. The production of terminal and solitary flowers in *LFY*-overexpressing plants is caused by the inhibition of *TFL1* expression induced by *LFY*^49^. *TFL1* expression was reduced in transgenic L12 plants (Supplementary Fig. S3I). Overexpression of *JcLFY* in the *Arabidopsis lfy-15* mutant recovered the late flowering phenotype, rescued the abnormal flowers of *lfy* mutant plants, and repressed inflorescence development (Fig. 3B–E). This suggests that *JcLFY* acted as a functional homolog of *LFY* in *Arabidopsis*.

### *JcLFY* expression was not induced by GA

GA has been generally found to strongly inhibit flowering in some woody perennial plants, such as citrus, rose, grape, and apple^36^,^50^,^51^,^52^. However, GA accelerates flowering in *Arabidopsis* and *Chrysanthemum* by inducing expression of the FM identity gene *LFY*^34^,^35^,^36^,^44^. We detected *JcLFY* expression levels in GA-treated shoot apex and flower bud tissues at different time points, revealing that *JcLFY* expression levels were not induced by GA. In contrast, *JcLFY* expression levels decreased between 3 and 48 h after GA application (Fig. 1E). This contrasts with *Arabidopsis* findings, suggesting GA may play a negative function in regulating flowering in *Jatropha*. Recently research revealed that although GA promoted termination of vegetative development, it inhibited flower formation in *Arabidopsis*^36^. Ghosh *et al*.^9^ found that paclobutrazol, a GA biosynthesis inhibitor, promotes flower initiation in *Jatropha*, further suggesting GA inhibits flower initiation in *Jatropha*. We propose that GA inhibition of flowering in *Jatropha* might occur via repression of *JcLFY* expression. This hypothesis need to be tested in future studies by determination of endogenous GA levels in different developmental stages of wild-type plants, and by analysis of phenotypic changes in flowering time in transgenic plants with altered GA biosynthesis or perception.

### *JcLFY* regulated flowering time and floral organs

*LFY* is a FM identity gene that plays an important role in promoting flowering in *Arabidopsis*^13^,^19^. The *JcLFY*-overexpressing transgenic *Arabidopsis* bolted two months earlier than WT plants under SD conditions (Table 2). However, *JcLFY* transgenic *Jatropha* plants took more than seven months to produce flower buds, although two months earlier than WT plants (Fig. 4). And in a subtropical area, the 35S:*JcLFY* transgenic *Jatropha* produced flowers until two or three years after plantation when no flower was found in WT plants (Supplementary Fig. S6). Compared to the 35S:*LFY* transgenic citrus described by Peña *et al*.^31^, the flowering time of transgenic *Jatropha* was very late; in citrus, transgenic shoots flowered just five weeks after regeneration. Therefore, the *JcLFY*-overexpression transgenic lines used in this work showed a slightly early flowering, but weaker phenotype than plants overexpressing the *Arabidopsis LFY* gene in *Arabidopsis*^27^ and citrus^31^. The early flowering phenotype of *JcLFY* transgenic *Jatropha* was also weaker than observed in studies of transgenic *Jatropha* plants overexpressing the florigen gene *JcFT*^41^,^42^, in which flower buds initiated directly from transformed callus 7 weeks after *in vitro* culture^47^. However, the *JcLFY* co-suppressed transgenic *Jatropha* plants did not exhibit late flowering (Fig. 5). These analysis of flowering time in overexpression and co-suppression plants suggest that *JcLFY* may not be a key flowering promoter.

In *Jatropha*, FMs are derived from inflorescence meristems (IMs), but FMs execute a developmental program very different from those of IMs. Thus, there must be factors that promote the determination of FMs but not IMs. *JcLFY* is one of these factors because co-suppression of *JcLFY* delayed flower formation, leading to production of more secondary inflorescence branches (Fig. 6D). Inactivation of *JcLFY* induced abnormal flower development in many aspects. First, the inflorescences and flowers were subtended by bracts (Fig. 5F,H). Second, most flowers were aborted, especially in the strongly co-suppressed plants. Third, the outermost floral organs were sepal-like. These sepaloid organs substantially outnumbered sepals and petals combined in WT plants (Fig. 7A–H). Fourth, the number of stamens was reduced, and the morphology of stamens was changed (Figs 6K,L and 7G,H). Fifth, carpels occurred in every flower of the co-suppressed plants, but their morphologies were abnormal, with irregular shapes and sizes (Figs 6G,H,K and 7C,D,G,H), which may result in the abnormal fruits in *JcLFY* co-suppressed plants (Fig. 8). Sixth, the ovules and ovule cavities were partially or completely absent in the co-suppressed plants (Fig. 7I–P).

The flower phenotypes of *JcLFY* co-suppressed plants were different from those of *Arabidopsis* single mutants of *ap1*, *ap2*, *ap3*, *ag*, *gi*, or *sep*^53^,^54^,^55^. The co-suppressed plants produced more inflorescences, bracts and sepaloid organs, and exhibited reduced fertility, closely resembling *Arabidopsis lfy* mutants^14^. Our qRT-PCR analysis (Supplementary Fig. S5) suggest these phenotypes may be induced by downregulation of *JcAP1*, *JcAP3*, *JcAG*, *JcSEP1*, *JcSEP2*, and *JcSEP3* in *JcLFY* co-suppressed *Jatropha*.

## Materials and Methods

### Plant materials

Wild-type *Jatropha* plants were grown in the Xishuangbanna Tropical Botanical Garden (XTBG; 21°54′N, 101°46′E, 580 m in altitude) of the Chinese Academy of Sciences located in Mengla County, Yunnan Province, southwestern China. Young transgenic *Jatropha* plants were grown in a greenhouse. Mature transgenic *Jatropha* were planted in a field plot within the XTBG in 2014–2015. The field plot for transgenic *Jatropha* plantation was isolated by other horticultural plant species and fruit trees, such as Bauhinia, papaya, and grapefruit. The roots, stems, mature leaves, inflorescence buds, flower buds, male flowers, female flowers, and fruits of *Jatropha* were collected during the summer. All of the tissues collected for qRT-PCR were immediately frozen in liquid N_2_ and stored at −80 °C until use. The *Arabidopsis thaliana* WT and *lfy-15* mutant of the Columbia ecotype (Col-0) were obtained from The *Arabidopsis* Information Resource (TAIR) (http://www.arabidopsis.org/). *Arabidopsis* seeds were germinated on 1/2 Murashige and Skoog (MS) medium for one week. Then, the seedlings were transferred to peat soil in plant growth chambers maintained at 22 ± 2 °C under long-day (LD; 16 h light/8 h dark) or short-day (SD; 8 h light/16 h dark) conditions. Phenotype analysis was performed on homozygous (T2) *Arabidopsis* plants and heterozygous (T0) *Jatropha* plants. More than 20 plants were used for the characterization of each *Arabidopsis* genotype. The number of rosette leaves, branches, and time (days) between transfer to soil and the appearance of the first flower bud as well as plant heights were recorded. The aboveground tissues of 15-day-old *Arabidopsis* seedlings were harvested to analyze mRNA transcription levels.

### Cloning full length *JcLFY* cDNA

Total RNA was isolated from *Jatropha* flower buds using the silica particle method^56^. cDNA synthesis was performed by using the SMART^TM^ cDNA Library Construction Kit (Clontech, Mountain View, CA, USA) according to the manufacturer’s instructions.

Two pairs of primers (XT97/XT98 and XT99/XT100) were designed according to the conserved *LFY* sequences of other woody species: *Ricinus communis* (gi|255540036|), *Populus trichocarpa* (gi|224136347|), *Salix discolor* (gi|29423801|), *Mangifera* (gi|323650468|), *Dimocarpus longan* (gi|144686979|), and *Buddleja davidii* (gi|76495760|). A 552-bp *JcLFY* fragment was isolated from *Jatropha* cDNA by nested PCR. *JcLFY* full length cDNA was obtained by RACE-PCR with gene-specific primers (XT130 for *JcLFY* 5′ RACE, XT131 for *JcLFY* 5′ RACE nested PCR, XT132 for *JcLFY* 3′ RACE, and XT133 for *JcLFY* 3′ RACE nested PCR), which were designed based on the 552-bp *JcLFY* fragment. A detailed sequence alignment was performed using BLAST against the NCBI database.

### Sequence and phylogenetic analyses

Specific primers (XT134/XT135) were designed to obtain the full length cDNA and genomic DNA sequences of *JcLFY*. Genomic sequences were amplified from 30-ng samples of DNA, and 2 μl of cDNA was used as a template for amplifying *JcLFY* full length cDNA sequences. The amplified PCR products were subjected to 1% agarose gel electrophoresis. Purified PCR fragments were cloned into a T&A cloning vector (Promega, Madison, Wisconsin, USA) for sequencing. Sequence alignment was carried out using Vector NTI 11 software (Invitrogen, Carlsbad, USA). To determine the amino acid sequence, the alignment results were subjected to pairwise comparisons using DNAMAN 5.0 (Lynnon Biosoft, Quebec, Canada). A phylogenetic tree based on the protein sequences was constructed with MEGA 5.0^57^. A neighbor-joining phylogenetic tree was generated with MEGA 5.0 using a Poisson model with gamma-distributed substitution rates and 1000 bootstrap replicates.

### Vector construction and plant transformation

To characterize the function of *JcLFY*, the coding region of *JcLFY* was cloned into a derived pORE R4 vector (obtained from TAIR) driven by the cauliflower mosaic virus (CaMV) 35S promoter at the *Sma*I and *Sal*I sites of the pORE R4 vector. The construct was transformed into *Agrobacterium tumefaciens* strain EHA 105 via the freeze-thaw method^58^ and transformed into WT and *lfy-15* mutant *Arabidopsis* by the *Agrobacterium tumefaciens*-mediated floral dip transformation method^59^. Transgenic lines were selected on 1/2 MS medium containing 50 μg/ml kanamycin, and transformed seedlings were further identified by *JcLFY*-specific PCR analysis. Verified transgenic T2 generation seedlings were transplanted into pots and grown in different light conditions for further experiments. Transformation of *Jatropha* with the *Agrobacterium* strain EHA105 carrying the same construct was performed according to the protocol described by Fu *et al*.^60^. All transgenic plants were confirmed using genomic PCR and RT-PCR.

### Semi-quantitative PCR and real-time qPCR analyses

Total RNA was isolated from different tissues of *Jatropha* using the silica particles method^56^. Ten RNA samples from different *Jatropha* tissues were obtained, including roots, young leaves, mature leaves, flower buds, flowers, shoot apices, pedicels, fruits, endosperms, and embryos. Total RNA was extracted from frozen *Arabidopsis* tissues derived from 15-day-old WT plants, *lfy-15* mutant plants, WT transgenic plants (lines 8, 11, and 12, henceforth L8, L11, and L12), and *lfy-15* mutant transgenic plants harboring *JcLFY* (plants C1 and C4) using TRIzol reagent (Biocentury Transgene, Shenzhen, China). Total RNA samples of transgenic and WT *Jatropha* were isolated from the flower buds using the silica particles method^56^. First-strand cDNA was synthesized with the PrimeScript^®^ RT Reagent Kit with gDNA Eraser (TAKARA, Dalian, China). The cDNA templates of first-strand cDNA were diluted 5-fold with sterilized double-distilled water. qRT-PCR was performed using SYBR^®^ Premix Ex Taq™ II (TAKARA) on a Roche 480 Real-Time PCR Detection System (Roche, Mannheim, Germany). The primers employed for qRT-PCR and semi-quantitative PCR are listed in Supplementary Table S1. qRT-PCR was conducted with three independent biological replicates and three technical replicates for each sample. The data were analyzed using the 2^−ΔΔCT^ method described by Livak and Schmittgen^61^. RT-PCR was carried out as described by Brownie *et al*.^62^. The transcript levels of specific genes were normalized using *Jatropha JcActin1* or *Arabidopsis Actin2*.

### Gene array analyses

The 35S:*JcLFY* transgenic *Arabidopsis* were transplanted into soil under LD conditions. Total RNA was extracted from the aboveground tissues of 2-week-old soil-grown WT *Arabidopsis* and transgenic *Arabidopsis* using an RNeasy^®^ Plant Mini Kit (Qiagen, Hilden, Germany) according to the manufacturer’s instructions. Total RNA was quantified, and its quality was assessed using an Agilent 2100 Bioanalyzer (Santa Clara, CA, USA). Microarray analysis was performed using the *Arabidopsis* (V4, 4 × 44) Gene Expression Microarray, Design ID: 021169 (Agilent) containing 43,603 *Arabidopsis* gene probes and 1,417 Agilent control probes. A total of at least 1 μg purified RNA was required for gene array analyses.

Labeled cRNA probes were fragmented using fragmentation buffer and hybridized to the *Arabidopsis* arrays in the presence of the Gene Expression Hybridization buffer HI-RPM and blocking agent for 17 h at 65 °C with a 10-rpm rotation speed in a hybridization oven. After the 17 h incubation, the arrays were washed using low stringency wash buffer 1 at room temperature for 1 min followed by a high stringency wash using wash buffer 2 at 37 °C. The arrays were air-dried and scanned using a high-resolution array scanner (Agilent) with the appropriate settings for one-color gene expression arrays. The signal intensities were extracted from the scanned images with the aid of Feature Extraction software 10.7.1.1 (Agilent) and subjected to background subtraction and spatial detrending. The outliers and abnormal features were flagged, and the data were normalized using intra-array percentile shift normalization (minimum threshold of 75) and median-based inter-array normalization. GeneSpring GX (Agilent) was used to calculate intensity ratios and fold changes. All genes with a *P*-value below 0.05 and at least 2-fold expression changes were chosen for a Gene Ontology enrichment analysis. The gene array hybridization experiments were performed by Shanghai Biotechnology Corporation (Shanghai, China).

### Analysis of flower, fruit, and seed phenotypes

One-year-old T0 transgenic plants grown in the Xishuangbanna Tropical Botanical Garden (XTBG; 21°54′N, 101°46′E, 580 m in altitude) of the Chinese Academy of Sciences were used to analyze the flower, fruit, and seed phenotypes. The seeds were harvested in autumn 2015. Flower and fruit anatomies were examined and photographed with a light Leica DM IRB anatomical lens (Leica, Heerbrugg, Switzerland) equipped with a Leica DFC425 C camera (Leica).

## Additional Information

**How to cite this article**: Tang, M. *et al*. An ortholog of *LEAFY* in *Jatropha curcas* regulates flowering time and floral organ development. *Sci. Rep*. **6**, 37306; doi: 10.1038/srep37306 (2016).

**Publisher’s note:** Springer Nature remains neutral with regard to jurisdictional claims in published maps and institutional affiliations.

## Supplementary Material

Supplementary Information

Supplementary Table S3

## Figures and Tables

**Table 1:** Overexpression of *JcLFY* promotes flowering in *Arabidopsis* under LD conditions.

Lines	N	Rosette leaves	Flower bud formation time/Day	Flowering time/Day	Height/cm	Branches	Cauline leaves
WT	22	12.36 ± 1.56	32.27 ± 3.87	40.59 ± 3.78	21.82 ± 2.52	4.14 ± 0.83	4.09 ± 0.61
Line8	17	10.41 ± 1.06*	23.24 ± 2.59**	32.59 ± 3.66**	18.06 ± 2.97*	4.06 ± 0.97	4.06 ± 1.39
Line11	16	9.25 ± 1.24*	19.43 ± 3.72**	26.94 ± 3.60**	16.16 ± 4.13*	2.25 ± 1.06**	3.38 ± 1.36
Line12	16	6.19 ± 1.11**	17.19 ± 2.83**	25.19 ± 1.94**	4.47 ± 1.59**	0.00 ± 0.00**	4.13 ± 1.15

WT plants and three independent *JcLFY*-overexpressing lines (L8, L11 and L12) grown under LD conditions (16 h light/8 h dark) were subjected to the analysis of rosette leaves, Flower bud formation time, flowering time, height, branches, and cauline leaves. N = plant number. The values are presented as the mean ± standard deviation. *Significantly different from the control at the 5% level; **significantly different from the control at the 1% level.

**Table 2:** Overexpression of *JcLFY* promotes flowering in *Arabidopsis* under SD conditions.

Lines	N	Rosette leaves	Flower bud formation time/Day	Flowering time/Day	Height/cm
WT	10	50.58 ± 5.14	126.50 ± 5.77	138.70 ± 14.47	38.88 ± 2.98
Line8	7	34.28 ± 4.22**	92.57 ± 8.62**	118.00 ± 11.76*	32.71 ± 7.96*
Line11	15	25.58 ± 3.49**	79.56 ± 6.69**	97.33 ± 10.13**	27.22 ± 2.90**
Line12	12	14.85 ± 2.07**	54.25 ± 3.70**	60.33 ± 4.52**	6.17 ± 3.29**

WT plants and three independent *JcLFY*-overexpressing lines (L8, L11 and L12) grown under SD conditions (8 h light/12 h dark) were subjected to the analysis of rosette leaves, flower bud formation time, flowering time, and height. N = plant number. The values are presented as the mean ± standard deviation. *Significantly different from the control at the 5% level; **significantly different from the control at the 1% level.

**Table 3:** Overexpression of *JcLFY* in *lfy-15 Arabidopsis* plants promotes flowering under LD conditions.

Lines	N	Rosette leaves	Flower bud formation time/Day	Flowering time/Day	Height/cm	Branches	Cauline leaves
WT	18	10.50 ± 2.09	25.39 ± 6.00	37.39 ± 6.10	31.11 ± 8.98	6.78 ± 1.90	5.78 ± 1.26
*lfy-15*	17	17.79 ± 3.36	35.18 ± 8.50*	51.71 ± 10.35**	32.47 ± 3.94	32.71 ± 7.30**	35.18 ± 9.02**
C1	16	8.06 ± 1.18**	17.19 ± 2.20**	23.31 ± 3.54**	7.31 ± 2.36**	0.00 ± 0.00**	5.62 ± 1.09
C4	15	4.36 ± 1.21**	12.46 ± 1.57**	18.23 ± 2.09**	5.25 ± 1.77**	0.00 ± 0.00**	4.79 ± 0.88

WT plants, the *lfy-15* mutant, and two independent *JcLFY*-overexpressing lines (C1 and C4) grown under LD growing conditions (16 h light/8 h dark) were subjected to the analysis of rosette leaves, flower bud formation time, flowering time, height, branches, and cauline leaves. N = plant number. The values are presented as the mean ± standard deviation. *Significantly different from the control at the 5% level; **significantly different from the control at the 1% level.
