# Supplementary material for: An ortholog of LEAFY in Jatropha curcas regulates flowering time and floral organ development
Source: Sci Rep. 2016 Nov 21;6:37306. doi: 10.1038/srep37306 (PMC5116762; doi:10.1038/srep37306)
Supplement: Supplementary Information [file srep37306-s1.docx]

**An ortholog of *LEAFY* in *Jatropha curcas* regulates flowering time and**

**floral organ development**

Mingyong Tang^1,2^, Yan-Bin Tao^1^, Qiantang Fu^1^, Yaling Song^1^, Longjian Niu^1^, Zeng-Fu Xu^1,^*

**Supplementary Table S1**: List of primers used in this study. F = forward primer; R = reverse primer.

| Gene | Primer name | Sequence (5′→ 3′) |
| --- | --- | --- |
| *AtActin2* qRT-PCR | XK718 F | TGTGCCAATCTACGAGGGTTT |
|  | XK719 R | TTTCCCGCTCTGCTGTTGT |
| *AtAG* qRT-PCR | XT898 F | GCGTCAACAAATAATCAGCATAC |
|  | XT899 R | CGAAGAATCTGGTTATCGTTATG |
| *AtAP1* qRT-PCR | XT803 F | TTTGGAGAGAAACCAGAGGCATT |
|  | XT804 R | GTAAGGATGCTGGATTTGGTGCT |
| *AtAP3* qRT-PCR | XT896 F | GTCTTGAGGATGAAATGGAAAAC |
|  | XT897 R | TGGTATCCAAGAACTGAGTCGTA |
| *AtBFT* qRT-PCR | XA362 F | GTGAGATACGAAACGCCTAAA |
|  | XA363 R | TAGACAGCAGCAACAGGTAGAG |
| *AtCAL* qRT-PCR | XT904 F | GGAGAGAAACCAAAGGCATTATC |
|  | XT905 R | TCCTTTCTTTGGAGGTGGTTGA |
| *AtFLC* qRT-PCR | XT900 F | CTTTCTGTTCTCTGTGACGCATC |
|  | XT901 R | AGTCTCAAGGTGTTCCTCCAGTT |
| *AtFT* qRT-PCR | XK674 F | GAACAACCTTTGGCAATGAGA |
|  | XK675 R | TCTTCCTCCGCAGCCACT |
| *AtLFY* qRT-PCR | XT805 F | TGCTCTCTCCCAAGAAGGGTTAT |
|  | XT806 R | TTGGTTTCTTTCTCCGTCTCTGC |
| *AtSEP1* qRT-PCR | XK904 F | CTTCTTGGGGAGGATTTAGGA |
|  | XK905 R | ACATTCTGTTCACCACCTTCC |
| *AtSEP2* qRT-PCR | XK906 F | GGACATCCTCAGGCTCATTCTC |
|  | XK907 R | AGAAGTATCGCTCACAGCATCC |
| *AtSEP3* qRT-PCR | XK908 F | AATGGGAAGAGGGAGAGTAGA |
|  | XK909 R | TTCTGGTGCTCCATAGTTACA |
| *AtTFL1* qRT-PCR | XT906 F | ATAATGGGGAGAGTGGTAGGAGA |
|  | XT907 R | TCTGGGTCTATCATCACCAAAGT |
| *AtTSF* qRT-PCR | XA360 F | ACCTGCCACCACTGGAAATG |
|  | XA361 R | CAGCCACAGGAAGACCAAGA |
| *Jcactin1* qRT-PCR | XK191 F | CTCCTCTCAACCCCAAAGCCAA |
|  | XK192 R | CACCAGAATCCAGCACGATACCA |
| *JcAG* qRT-PCR | XK658 R | TGTGGATAATGATTGGTGGGTTG |
|  | XK659 F | TCTGATGCCAGGAGGAGGTAACT |
| *JcAP1* qRT-PCR | XA311 F | TAACAGACTCAAGGCGAAGGT |
|  | XA312 R | AGTTGGTTGTTTCTTGCTCGG |
| *JcAP2* qRT-PCR | XB715 F | GGTGAGAATGATGACCGTAGA |
|  | XB716 R | AACTGAATTTGATAACTGGGTG |
| *JcAP3* qRT-PCR | XT374 F | CTCTTGGAATAAGTAACCTGTCTGTTGG |
|  | XT375 R | CAAAACCCACTACTACAAAACCGAAGA |
| *JcLFY* 3’RACE | XT132 F | GGTGGGTATGAGAGACAGCGGGAGC |
|  | XT133 F | GAGGAAAGGGCAGAGGAAGGCGGTC |
| *JcLFY* 5’RACE | XT130 R | CTTGTTGATGTAGCTCGCTCCTGCC |
|  | XT131 R | CCTGAACACCTGATTCGTCACCTTG |
| *JcLFY* full length | XT134 F | CGAGCTCGATAGCAGCAAAATCTTCAGTTCAAC |
|  | XT135 R | GCGTCGACTCCTAACCTACAACAATAAACAAAC |
| *JcLFY* nest | XT99 F | AGAGGAGCCAGTGCAGCAAGAGAAG |
|  | XT100 R | CTCCATGCTCCAACATTTTCCCCTC |
| *JcLFY* partial | XT97 F | TAGGTGGGATCTTCTTGTTGGGGAG |
|  | XT98 R | GGGGATGTGCATTGAAAATAGCG |
| *JcLFY* qRT-PCR | XT655 F | GGATAAGATACTACACAGCAGCGA |
|  | XT656 R | TAACCCTTCTTGAGAGAGAGCATC |
| *JcSEP1* qRT-PCR | XA945 F | GCGATGCTGAAGTTGCTCTCATTATCT |
|  | XA946 R | CTCCTGGTAGTTGCCCTGTGATTC |
| *JcSEP2* qRT-PCR | XA947 F | GCTTTAAGGAAGAAGTGGGAGGAAACAA |
|  | XA948 R | TGAATGAGGAGGCTGGCAGTTG |
| *JcSEP3* qRT-PCR | XA949 F | CCTCAGGGCGATGGTTTCTTTCA |
|  | XA950 R | TCACTACTGTTATTGGATCTGGCTGGTA |
| *JcSOC1* qRT-PCR | XK656 F | TTCTTGGACGGCAACGCTTA |
|  | XK657 R | CTCTCGGAAAAGTGTGGGATC |
| *JcTFL1a* qRT-PCR | XA520 F | GTGTATGTTAGTACCGTATTTGGAT |
|  | XA521 R | CTAAACCAAAGAGCTTATTCTAGGC |
| *JcTFL1b* qRT-PCR | XA485 F | ACCAGTAGACCCTCTTATTGTTGAGA |
|  | XA486 R | TCATATCATCTCCTTCCACAGCAACT |
| *JcTFL1c* qRT-PCR | XA203 F | ACGGAGCCACAGCCACTTACTGTAG |
|  | XA204 R | ACTCTAGGTTTAGCAGCAATGACCG |

**Supplementary Table S2:** Fold changes (FCs) of the expression of the potential JcLFY target genes in transgenic *Arabidopsis* lines L8, L11, and L12.

| No. | GenBank  identifier | GenBank predicted activity | Functional category | Line 8 FC | Line 11 FC | Line 12 FC | *AtLFY* overexpression FC^R^ |
| --- | --- | --- | --- | --- | --- | --- | --- |
| 1 | AT5g60630 | Signaling protein | Signaling | **4.5** | **1.1** | **5.5** | **46** |
| 2 | AT5g49770 | Leucine-rich repeat receptor kinase | Signaling | **1.5** | **-1.8** | **1.2** | **27** |
| 3 | At5g03790 | Homeodomain transcription factor | Transcription | **1.5** | **2.0** | **1.0** | **11** |
| 4 | At3g61250 | myb DNA-binding protein (MYB17) | Transcription | **5.5** | **2.9** | **5.3** | **5** |
| 5 | At1g16070 | Tubby-related transcription factor | Transcription | **3.7** | **3.3** | **1.8** | **4** |
| 6 | AT4g14090 | UDP-glycosyltransferase | Protein modification | **2.2** | **2.8** | **-2.6** | **3** |
| 7 | AT5g03230 | HMG motif-containing DNA binding | Transcription | **1.6** | **1.4** | **2.3** | **3** |
| 8 | At4g22780 | EF-1 α -like | Translation | **1.0** | **0.95** | **1.0** | **3** |
| 9 | At3g43190 | Sucrose synthase | Sugar /amino acid biosynthesis | **4.2** | **2.8** | **-1.8** | **3** |
| 10 | AT3g52470 | Similar to HIN1 protein | Signaling | **-1.1** | **-1.7** | **-1.3** | **3** |
| 11 | At3g47340 | Glutamine-dependent asparagine synthetase | Sugar/amino acid biosynthesis | **2.6** | **1.8** | **1.8** | **2.5** |
| 12 | AT5g46660 | CHP-rich zinc finger protein | Transcription | **-1.1** | **1.0** | **1.0** | **2.5** |
| 13 | At2g44450 | Glycosyl hydrolase family 1 | Sugar/amino acid biosynthesis | **2.0** | **1.3** | **1.0** | **2.5** |
| 14 | AT3g19390 | Cysteine proteinase | Protein modification | **-2.8** | **-2.6** | **-1.7** | **2** |
| 15 | At1g68880 | bZIP family transcription factor | Transcription | **1.0** | **6. 9** | **1.0** | **2** |

^R^ the results referred from William, D. A. et al. Genomic identification of direct target genes of LEAFY. *PNAS* **101**, 1775-1780, doi:10.1073/pnas.0307842100 (2004).

**Supplementary Table S3** Classification of the genes with a differential expression between wild-types (WT) and transgenic *Arabidopsis* lines (L8, L11, and L12) exhibiting fold changes (FCs) greater than 2. (Please refer to a separate Excel file)

**
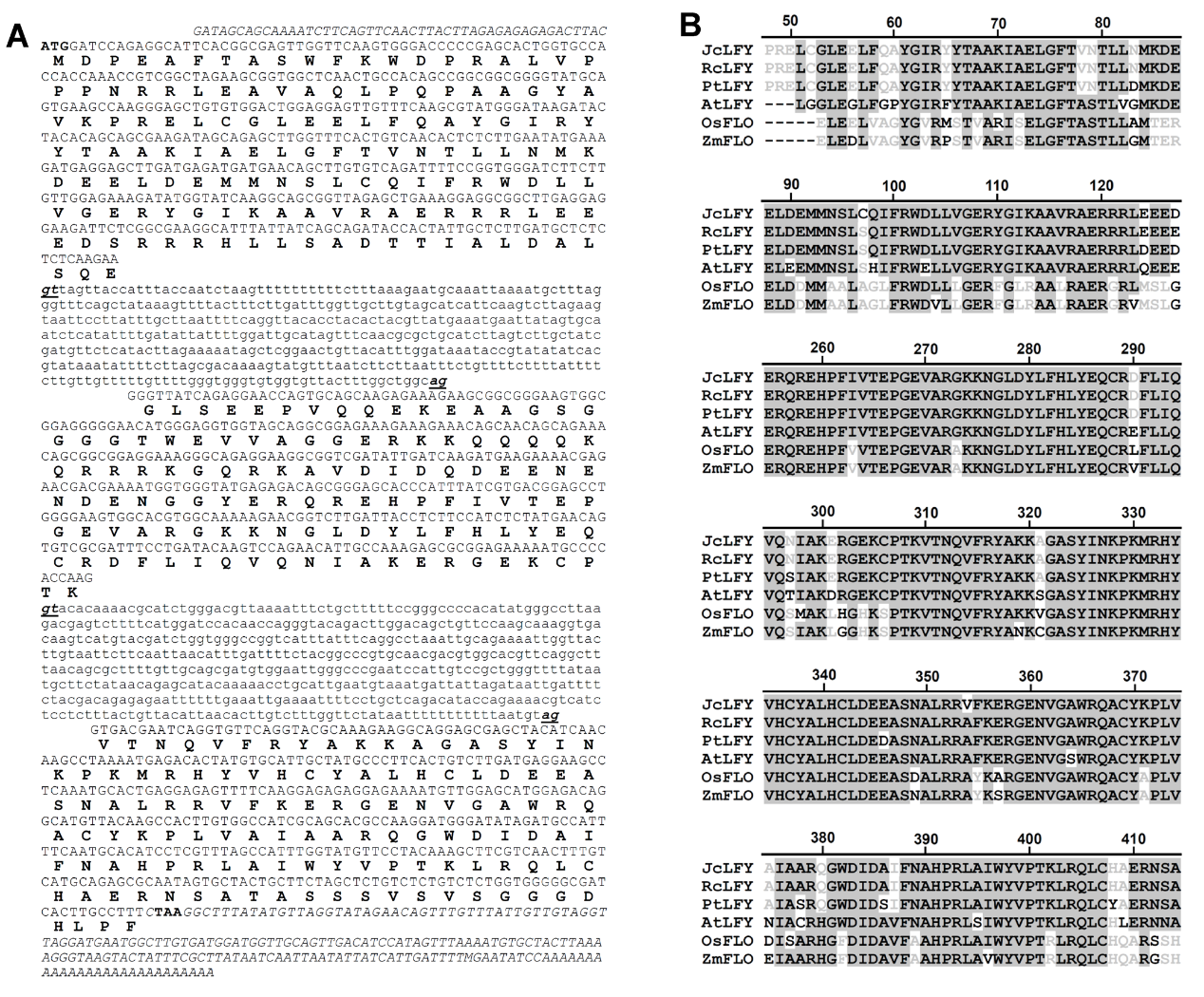
**

**Supplementary Figure S1: JcLFY sequence information and alignment of *LFY* homologs.** (A) *JcLFY* gDNA sequence, cDNA sequence, and amino acid sequence. The introns are shown in lowercase letters, while cDNA sequences are shown in capital letters, amino acids are shown in bold capital letters, and untranslated regions are shown in italic capital letters. (B) Alignment of translated cDNA sequence of *Jatropha curcas* LFY (JcLFY), *Ricinus communis* LFY (RcLFY), *Populus trichocarpa* LFY (PtLFY), *Arabidopsis thaliana* LEAFY (AtLFY), *Oryza sativa* FLO (OsFLO), and *Zea mays* FLO (ZmFLO).


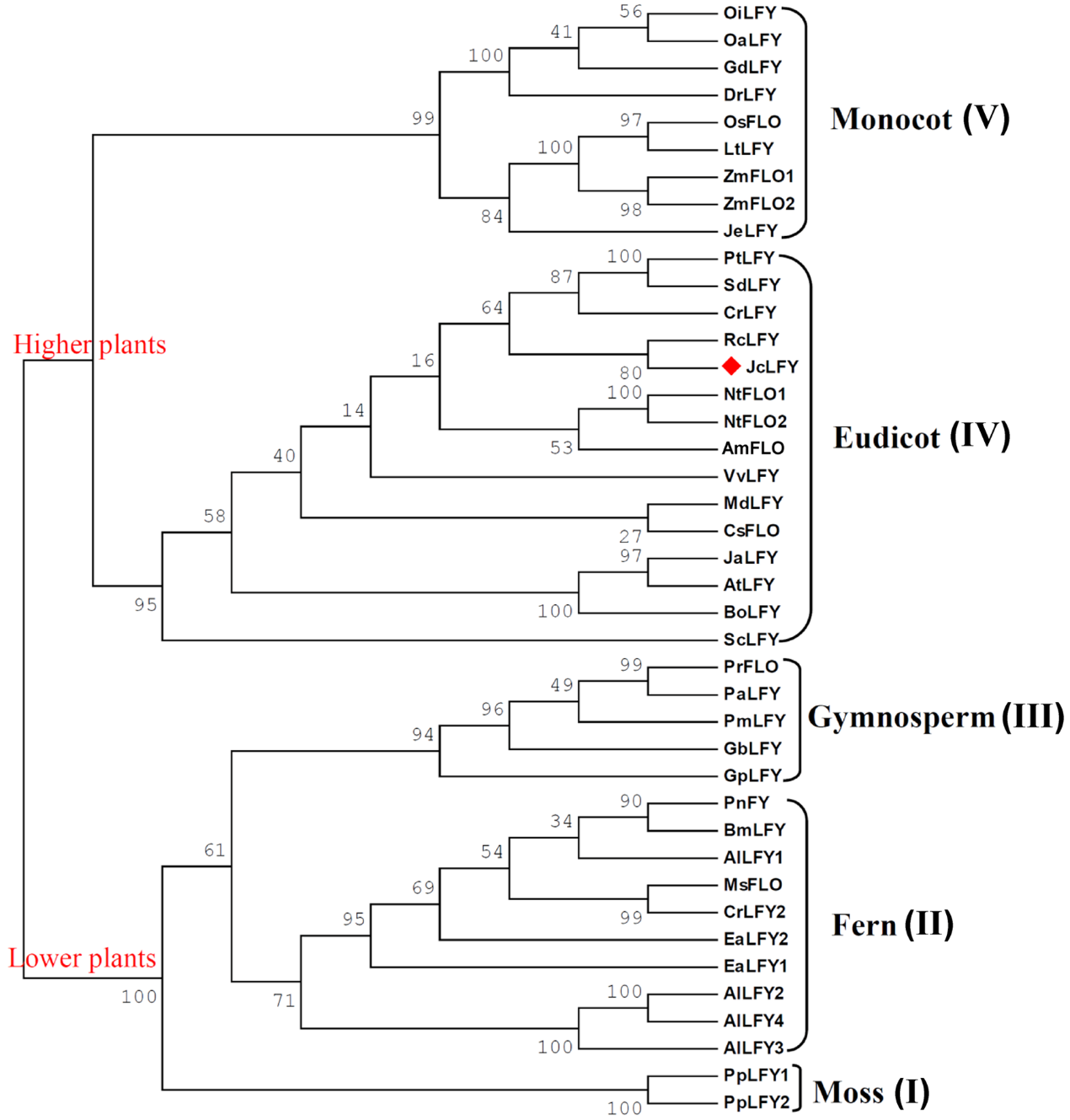


**Supplementary Figure S2: Unrooted neighbor-joining tree of LFY using MEGA 5.0.** Branches with bootstrap support (1000 replicates) exceeding 50% are indicated. The phylogenetic tree of JcLFY amino acid sequences was constructed as described in the Materials and methods. The following amino acid sequences were obtained from GenBank: XP_002511083 for *Ricinus communis* (RcLFY), AAO53547 for *Populus trichocarpa* LFY (PtLFY), AAO73539 for *Salix discolor* LFY (SdLFY), ABJ97283 for *Citrus reticulata* LFY (CrLFY), Q40504 for *Nicotiana tabacum* LFY1 (NtFLO1), Q40505 for *Nicotiana tabacum* LFY2 (NtFLO2), P23915 for *Antirrhinum majus* floricaula (AmFLO), XP_002284664 for *Vitis vinifera* LFY (VvLFY), BAD10949 for *Malus* x *domestica* LFY (MdLFY), AAC64705 for *Cucumis sativus* LFY (CsFLO), AAF00503 for *Jonopsidium acaule* LFY (JaLFY), Q05536 for *Brassica oleracea* LFY (BoLFY), AAM27927 for *Arabidopsis thaliana* LFY (AtLFY), BAA21547 for *Oryza sativa* FLO (OsFLO), NP_001105201 for *Zea mays* FLO1 (ZmFLO1), AAV68220 for *Zea mays* FLO2 (ZmFLO2), AAG41992 for *Lolium temulentum* LFY (LtLFY), AAF77077 for *Juncus effusus* LFY (JeLFY), BAC54955 for *Orchis italica* LFY (OiLFY), BAC55072 for *Orchis anthropophora*LFY (OaLFY), BAC55083 for *Dactylorhiza romana* LFY (DrLFY), BAC55084 for *Gennaria diphyll* LFY (GdLFY), AAF77075 for *Ginkgo biloba* LFY (GbLFY), O04116 for *Pinus radiate* FLO (PrFLO), BAA93436 for *Gnetum parvifolium* LFY (GpLFY), AAV49504 for *Picea abies* LEAFY (PaLFY), AAY35059 for *Podocarpus matudae* LFY (PmLFY), BAB88867 for *Angiopteris lygodiifolia* LFY1 (AlLFY1), BAB88868 for *Angiopteris lygodiifolia* LFY2 (AlLFY2), BAB88869 for *Angiopteris lygodiifolia* LFY3 (AlLFY3), BAB93543 for *Angiopteris lygodiifolia* LFY4 (AlLFY4), BAB88866 for *Equisetum arvense* LFY1 (EaLFY1), BAB90844 for *Equisetum arvense* LFY2 (EaLFY2), AAF77608 for *Matteuccia struthiopteris* FLO (MsFLO), BAB41070 for *Ceratopteris richardii* LFY2 (CrLFY2), BAB88863 for *Psilotum nudum* LFY (PnLFY), BAB88864 for *Botrychium multifidum* LFY (BmLFY), XP_001762054 for *Physcomitrella patens* LFY1 (PpLFY1), XP_001765301 for *Physcomitrella patens* LFY2 (PpLFY2), CAC86163 for *Silene* *coeli-rosa* SilLFY (ScLFY). This phylogenetic tree was constructed based on LFY amino acid sequences from forty plant species. The tree was constructed by the neighbor-joining method, and bootstrapped 1000 times in order to estimate the support for internal nodes. Each ortholog group is clustered within its own branch. The species names included in this figure are abbreviated.


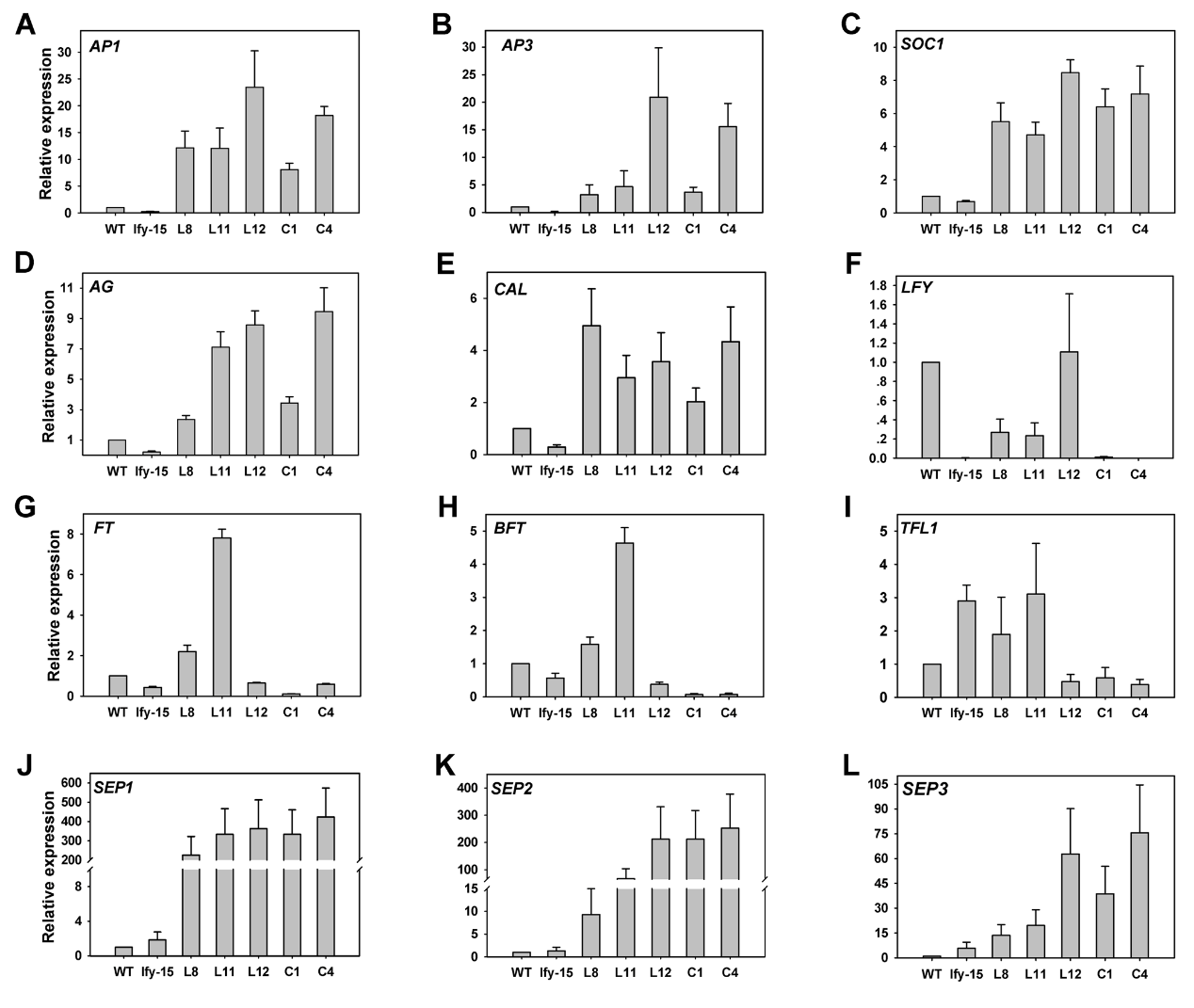


**Supplementary Figure S3: Real-time qRT-PCR analysis of expression of the flower-related genes in transgenic *Arabidopsis*.** Real-time qRT-PCR analysis was performed on wild-type (WT), *lfy-15* mutant, L8, L11, and L12 plants as well as C1 and C4 plants, which were *lfy-15* mutants harboring 35S:*JcLFY*. The expression abundance of *AP1*, *AP3*, *SOC1*, *AG*, *CAL,* *LFY*, *FT*, *BFT*, *TFL1*, *SEP1*, *SEP2*, and *SEP3* were individually assayed*.* RNA samples were extracted from 15-day-old seedlings of the 35S:*JcLFY* transgenic, WT, and *lfy-15* mutant *Arabidopsis*, which were grown in pots under LD conditions. Transcript levels were normalized using *AtActin2* as a reference gene. The mRNA level in WT plants was standardized as having a value of 1.


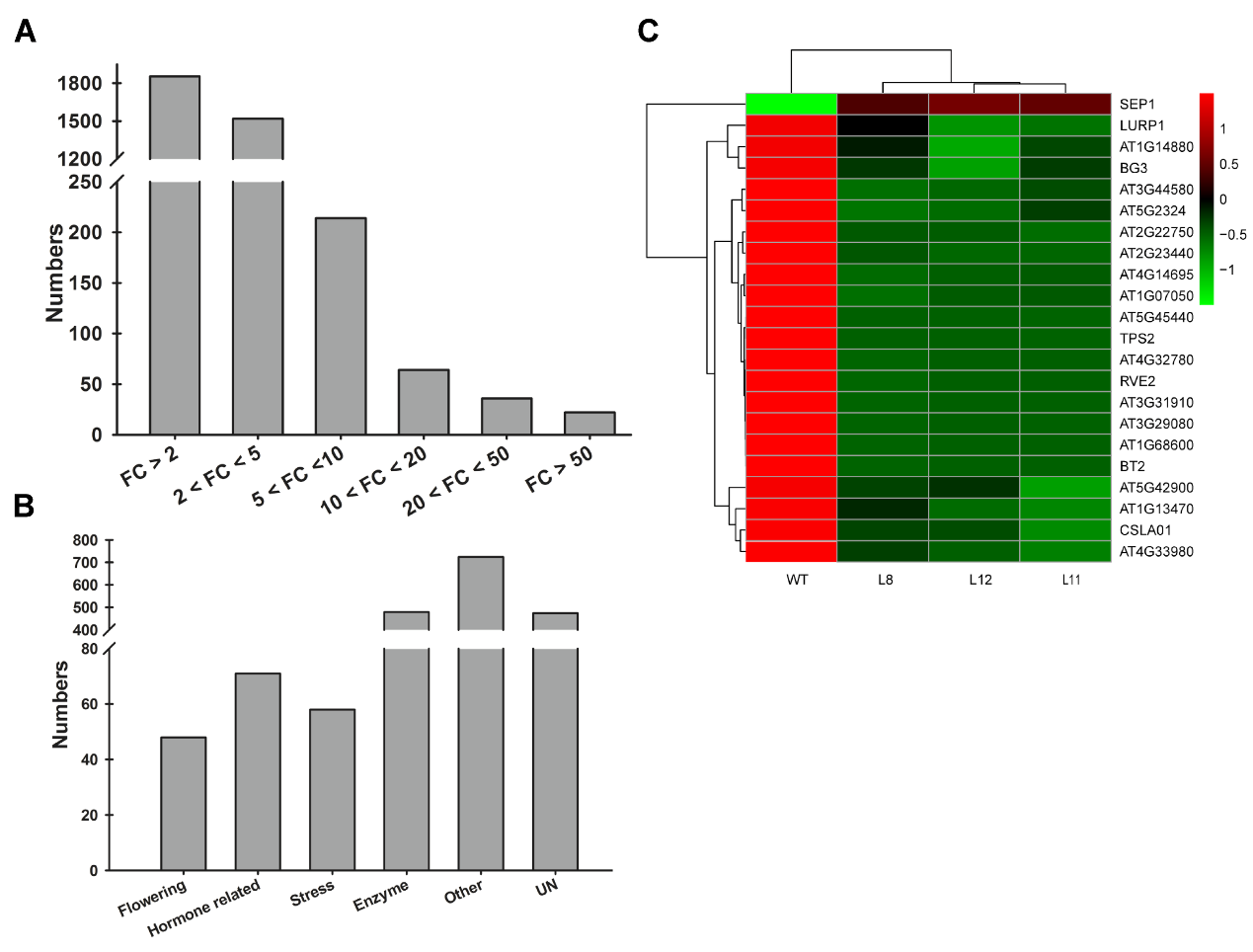


**Supplementary Figure S4: Analysis of microarray data.** (A) All genes were classified according to the fold change [test vs. wild-type (WT)]. WT plants were cultured in the same conditions as controls. “FC > 2” is the number of genes for which fold changes (FCs) were greater than 2 in analyzed transgenic *Arabidopsis* (from L8, L11, and L12); “2 < FC < 5” is the number of genes for which FCs were greater than 2, but less than 5; “5 < FC < 10” is the number of genes for which FCs were greater than 5, but less than 10. “10 < FC < 20” is the number of genes for which FCs were greater than 10, but less than 20. “20 < FC < 50” is the number of genes for which FCs were greater than 20, but less than 50. “FC > 50” is the number of genes for which FCs were greater than 50. (B) FCs more than 2 are divided into 6 groups, including flowering-related, hormone pathway-related, stress, metabolism-related enzymes, other (corresponding to all gene functions that do not belong to the other groups), and UN (gene functions are unknown). (C) Clustering of genes with FCs more than 50.


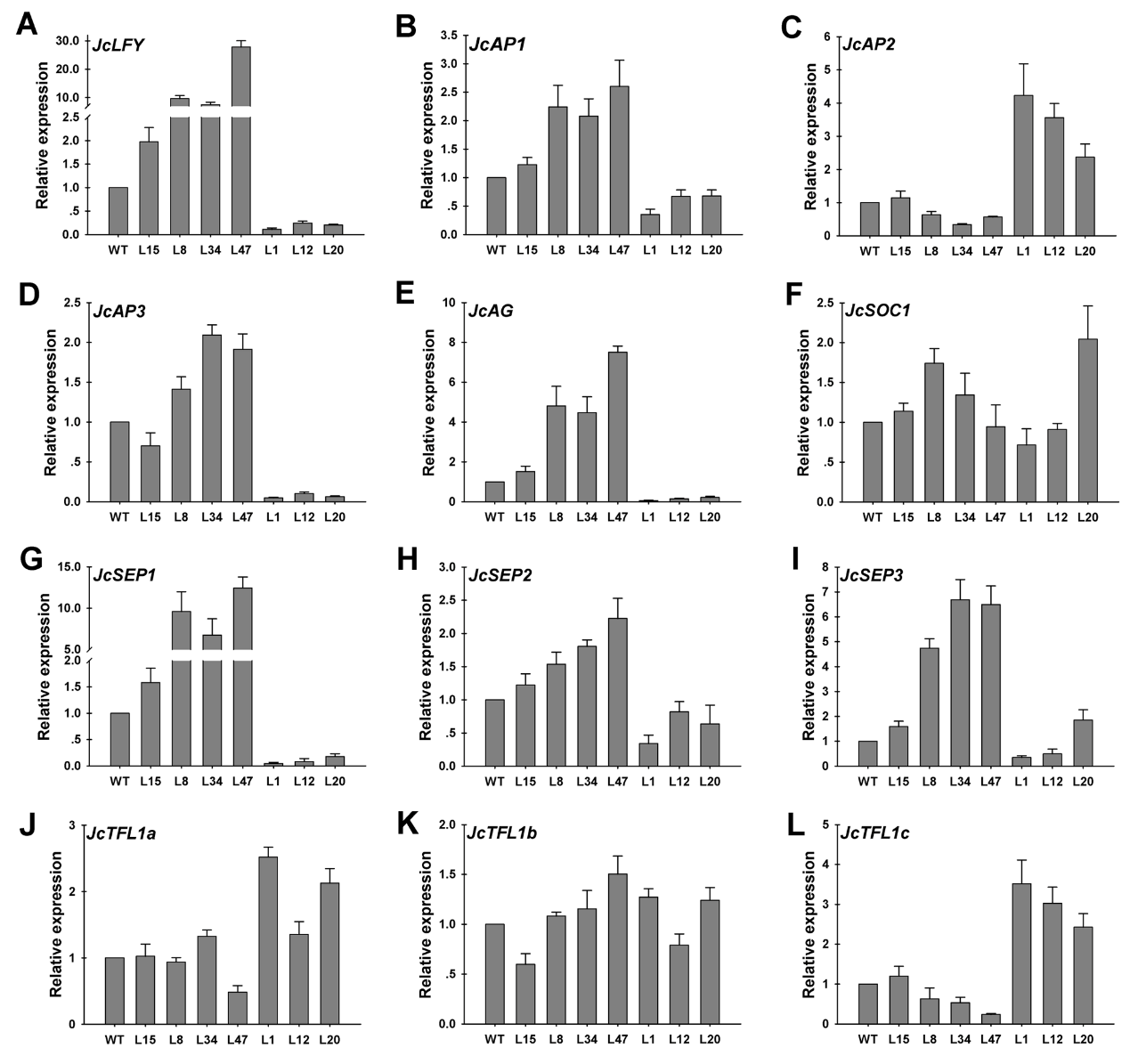


**Supplementary Figure S5: Real-time qRT-PCR analysis of expression of the flowering-related genes in transgenic *Jatropha*.** Real-time qRT-PCR analysis performed on wild-type (WT), overexpression lines (L15, L8, L34, and L47) and co-suppression lines (L1, L12, and L20) of *Jatropha*. The expression abundances of *JcLFY*, *JcAP1*, *JcAP2*, *JcAP3*, *JcAG, JcSOC1, JcSEP1*, *JcSEP2*, *JcSEP3*, *JcTFL1a*, *JcTFL1b*, and *JcTFL1c* are given*.* RNA samples were extracted from flower buds of 35S:*JcLFY* transgenic plants and WT plants. Transcript levels were normalized using *Jcactin1* as a reference gene. The mRNA level in WT was standardized as having a value of 1.


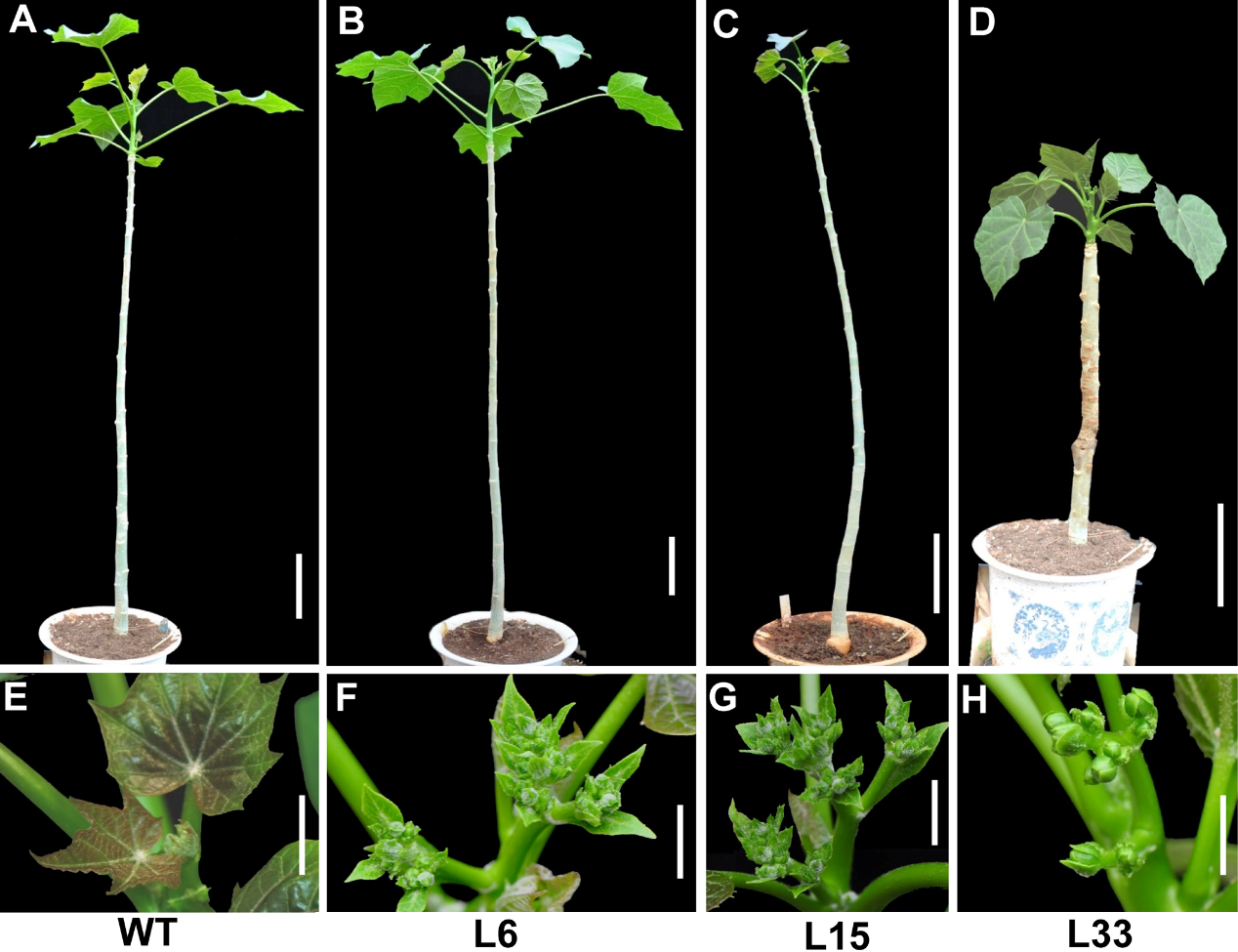


**Supplementary Figure S6: The 35S:*JcLFY* transgenic *Jatropha* produced flowers in a greenhouse during the second and third spring in a subtropical area (Kunming, China)**. (A) 3-year-old WT *Jatropha*; (B) 3-year-old 35S:*JcLFY* transgenic *Jatropha* plantlet (L6); (C, D) 2-year-old 35S:*JcLFY* transgenic *Jatropha* plantlet (L15, L33); (E) the shoot apex of the WT in (A), no flower buds were found; (F-H) The flower buds of 35S:*JcLFY* transgenic *Jatropha* in (B-D) respectively. (A-D) bar = 10 cm, (E-H) bar = 1 cm.
